# Multi-Omics Studies Demonstrate *Toxoplasma gondii*-Induced Metabolic Reprogramming of Murine Dendritic Cells

**DOI:** 10.3389/fcimb.2019.00309

**Published:** 2019-09-11

**Authors:** Kerrie E. Hargrave, Stuart Woods, Owain Millington, Susan Chalmers, Gareth D. Westrop, Craig W. Roberts

**Affiliations:** Strathclyde Institute of Pharmacy & Biomedical Sciences, University of Strathclyde, Glasgow, United Kingdom

**Keywords:** dendritic cells, immunometabolism, *Toxoplasma gondii*, LPS stimulation, Warburg effect, arginine metabolism, multi-omics

## Abstract

*Toxoplasma gondii* is capable of actively invading almost any mammalian cell type including phagocytes. Early events in phagocytic cells such as dendritic cells are not only key to establishing parasite infection, but conversely play a pivotal role in initiating host immunity. It is now recognized that in addition to changes in canonical immune markers and mediators, alteration in metabolism occurs upon activation of phagocytic cells. These metabolic changes are important for supporting the developing immune response, but can affect the availability of nutrients for intracellular pathogens including *T. gondii*. However, the interaction of *T. gondii* with these cells and particularly how infection changes their metabolism has not been extensively investigated. Herein, we use a multi-omics approach comprising transcriptomics and metabolomics validated with functional assays to better understand early events in these cells following infection. Analysis of the transcriptome of *T. gondii* infected bone marrow derived dendritic cells (BMDCs) revealed significant alterations in transcripts associated with cellular metabolism, activation of T cells, inflammation mediated chemokine and cytokine signaling pathways. Multivariant analysis of metabolomic data sets acquired through non-targeted liquid chromatography mass spectroscopy (LCMS) identified metabolites associated with glycolysis, the TCA cycle, oxidative phosphorylation and arginine metabolism as major discriminants between control uninfected and *T. gondii* infected cells. Consistent with these observations, glucose uptake and lactate dehydrogenase activity were upregulated in *T. gondii* infected BMDC cultures compared with control BMDCs. Conversely, BMDC mitochondrial membrane potential was reduced in *T. gondii*-infected cells relative to mitochondria of control BMDCs. These changes to energy metabolism, similar to what has been described following LPS stimulation of BMDCs and macrophages are often termed the Warburg effect. This metabolic reprogramming of cells has been suggested to be an important adaption that provides energy and precursors to facilitate phagocytosis, antigen processing and cytokine production. Other changes to BMDC metabolism are evident following *T. gondii* infection and include upregulation of arginine degradation concomitant with increased arginase-1 activity and ornithine and proline production. As *T. gondii* is an arginine auxotroph the resultant reduced cellular arginine levels are likely to curtail parasite multiplication. These results highlight the complex interplay of BMDCs and parasite metabolism within the developing immune response and the consequences for adaptive immunity and pathogen clearance.

## Introduction

*Toxoplasma gondii* is an obligate intracellular parasite that actively invades mammalian cells where it multiplies rapidly before actively egressing to infect other cells. It can infect almost any mammalian cell type including phagocytes such as dendritic cells (Dubey, 1998; Sibley, 2011). As an intracellular parasite *T. gondii* has evolved a close relationship with mammalian host cells and is known to rely on them for a number of nutrients (Silva et al., 2002; Dubey, 2004; Sibley et al., 2012). Conversely the host has evolved immune mechanisms that directly kill *T. gondii* or restrict the availability of certain nutrients and thus control the growth of the parasite (Denkers, 2010; Pifer and Yarovinsky, 2011). In addition, a considerable body of literature now recognizes that activation of phagocytes during infection not only results in alteration of canonical immune markers and mediators, but causes fundamental changes to metabolism with potential to direct the quality of the immune response. Therefore, the nature and combination of these early events in these cells may play a pivotal role in establishing *T. gondii* infection, initiating host immunity and determining the outcome of acute toxoplasmosis.

An important immune-mediated metabolic change known to occur in phagocytes is energy metabolism which is responsible for the production of ATP used in all energy dependent processes within cells (Hinkle and McCarty, 1978). In the most simplistic form, ATP can be formed through glycolysis in the cytosol and yields two ATP molecules. Pyruvate is made as a by-product of this process and in anaerobic conditions is fermented to lactate. In aerobic conditions, pyruvate can be fed into the TCA cycle located within the mitochondria to produce thirty-six ATP molecules. This is dependent on a functioning electron transport chain (ETC) and this entire process is termed, oxidative phosphorylation. Unsurprisingly, ~95% of a naïve immune cells' energy is generated via oxidative phosphorylation under normoxic conditions. However, it has been shown under certain circumstances, immune cells favor ATP production by glycolysis, even in an normoxic environment. This is known as aerobic glycolysis or the “Warburg effect” (Warburg et al., 1958; Reviewed by Palsson-McDermott and O'Neill, 2013). Changes in the TCA cycle can also affect a large variety of biosynthetic processes for which it provides essential intermediates. Thus, modulation of energy metabolism has proximal and distal functional benefits for immune cells including macrophages and dendritic cells as it provides the precursors for protein manufacture and fatty acid synthesis required for cytokine production and membrane remodeling.

The vast majority of work described thus far in the literature has used LPS, IFN-γ, or IL-4 as surrogate immune activators rather than live infections. Within the literature, metabolic reprogramming of LPS (innate activation), LPS + IFN-γ (classical activation) or IL-4 (alternative activation) treated macrophages has been well-documented (Pearce et al., 2013; Murray et al., 2014). Generally, in the literature, LPS stimulation is associated with numerous metabolic changes culminating in a shift from oxidative phosphorylation to aerobic glycolysis (“Warburg effect”). In contrast, IL-4 stimulation is associated with augmented oxidative phosphorylation and ATP production (Newsholme et al., 1986; O'Neill and Hardie, 2013; Jha et al., 2015). BMDCs are known to be responsive to many of the same mediators as macrophages and depending on their activation status, share a number of functions (O'Neill and Pearce, 2016). As the main antigen presenting cells in the body, DCs have the unique ability to initiate T cell activation by delivering antigen from the periphery to secondary lymphoid organs. In addition, DCs that encounter pathogens generate cytokines and chemokines attracting or activating other cell types at the site of infection (Austyn, 2016). *In vitro* granulcoyte (GM-CSF) differentiated BMDCs have been likened to *in vivo* murine iDCs (Guilliams et al., 2016) and studies have shown that stimulation with LPS induces increased glucose uptake, aerobic glycolysis and augmentation of the pentose phosphate pathway while suppressing oxidative phosphorylation (encompassing both the TCA and ETC) (Jantsch et al., 2008; Krawczyk et al., 2010). This leads to “enzymatic breaks” in the TCA cycle and intermediate accumulation which is used for other immune functions (Rubic et al., 2008; Infantino et al., 2011; Tannahill et al., 2013; Everts et al., 2014). In addition, a dichotomy exists between LPS stimulated and IL-4 treated BMDCs in terms of arginine metabolism. LPS stimulation of BMDCs, like macrophages, was found to promote increased arginine metabolism as determined by an increase in both inducible nitric oxide synthase (iNOS) and the production of citrulline and arginosuccinate. In contrast, IL-4 treatment induces the conversion of arginine into ornithine and proline via arginase (Corraliza et al., 1995; Munder, 1997; Van den Bossche et al., 2012; Reviewed by Thwe and Amiel, 2018).

Host cells recognize *T. gondii* through a number of pathogen associated molecular patterns (PAMPS) that are known to activate toll-like receptors (TLR2, 4 and 11) in mice and in theory have the ability to induce similar changes as seen with LPS stimulation of phagocytes (Butcher et al., 2005) Investigating how host cells reprogram their metabolism in response to intracellular pathogens is complex. Firstly, it is challenging to separate host from pathogen metabolism in many assays. Secondly, these pathogens have a number of secretory molecules that can also alter host cell metabolism for their own benefit. To overcome this, the transcripts can be aligned to the mouse genome and although there is a degree of similarity between host and parasite enzymes at the amino acid level, they are very different at the DNA level and can therefore be identified unambiguously. This strategy has been used previously for the study of a variety of different intracellular and extracellular pathogens (Blader et al., 2001; Chaussabel et al., 2003).

Herein, we used a multi-omics approach comprising transcriptomics and metabolomics on murine GM-CSF differentiated dendritic cells which we validated with functional studies to better understand how *T. gondii* metabolically reprograms dendritic cell metabolism *in vitro* and how this compares with the effect of LPS. We show that some observed changes are consistent with parasite evolved mechanisms to subvert the host immune response, while other are consistent with host derived mechanisms to control parasite dissemination.

## Materials and Methods

### Mice

Eight to ten-week-old, male BALB/c mice were bred and maintained in house at the Strathclyde Institute of Pharmacy and Biomedical Sciences, Glasgow, UK. All animal procedures including Schedule 1 conformed to guidelines from The Home Office of the UK Government.

### Generation of Murine Dendritic Cells

Bone marrow derived dendritic cells were cultured by flushing femurs and tibia of 8 to 10-week-old BALB/c mice with RPMI 1640 supplemented with 10% heat inactivated FCS, 10% Granulocyte-monocyte colony stimulating factor (conditioned from the supernatant of x63 cells), 5 mM L-glutamine, 100 U/ml penicillin, 100 μg/ ml streptomycin and incubated at 37°C for 7 days. After this time, adherent and semi-adherent cells were harvested and then seeded into either 24 well plates (1 × 10^6^ cells/ ml) or 96 wells plates (1 × 10^5^ cells/100 ul).

### Maintenance of Transfected *T. gondii* Prugniaud Strain

Tachyzoites were routinely maintained in confluent human foreskin fibroblasts (HFFs) grown in DMEM complete medium comprising of 10% fetal calf serum, 5 mM L-glutamine, 100 U/ml penicillin, 100 μg/ ml streptomycin and 50 U/ml amphotericin B at 37°C in 5% CO_2_.

### Dendritic Cell Infection With *T. gondii*

For infection, 1 × 10^6^ BMDCs were seeded into 24 well-plates in complete RPMI 1640 medium. The cells were infected with 1 × 10^6^ type II Prugniaud strain *T. gondii* tachyzoites. In some experiments, dendritic cells were also treated with *E.coli* LPS (1 μl/ml).

### Measuring Nitric Oxide in Cell Supernatant

Cell supernatant nitric oxide levels were determined by Griess assay. Cell supernatant or standards were added in equal volumes with Griess Reagent (1:1 ratio of 2% sulphanilamide in 5% H3PO_4_ and 0.2% Napthylene diamine HCL in ddH_2_O) in a 96 well-plate and incubated in the dark for 10 min. Absorbance was read at 540 nm on a Spectromax 190 plate reader and serum nitrite concentrations were calculated against a standard curve.

### Measurement of Arginase-1 Expression

Arginase activity was measured using an assay based on a reaction with α-isonitrosopropiophenon (ISPF) as described previously (Al-Mutairi et al., 2010). Briefly, BMDCs were grown on 24-well plates, exposed to agonist as appropriate and harvested in 50 ul lysis buffer (50 mM Tris-HCL, 10 mM MnCl2, 0.1% Triton X-100, 5 ug/ml pepstatin A, 5 ug/ml aprotinin, and 5 ug/ml antipain hydrochloride, pH 7.4). Arginine hydrolysis was carried out by incubating cell lyates with 25 ul of 0.5M L-arginine (pH 9.7) at 37°C for 60 min. The reaction was terminated by adding 400 ul of an acid solution (H2SO_4_, H3PO_4_, and H_2_O in a ratio of 1:3:7 and 25 ul of 9% solution of ISPF). Samples along with known urea standards were incubated at 95°C for 45 min and then allowed to cool for 10 min in the dark. Aliquots were added to wells of a 96 well-plate and absorbance read at 540 nm on a Spectromax 190 plate reader.

### Flow Cytometry

Cells were harvested for staining and incubated with Fc-block for 10 min at room temperature. Cells were stained for 1 h at 4°C with CD11c—PE or FITC, CD40—APC, CD80—FITC, CD86—APC-cy7 (BD bioscience). Cells were fixed using Fix & Perm (Life Technologies) following manufacturer's instructions. Intracellular staining was performed using iNOS—PE (ebioscience). For glucose uptake assays, 50 ul fluorescent glucose analog (2-NBDG-FITC) was added to the cells 120 min before harvest. CFSE labeled *T. gondii* was used for uptake assays. A total of 30, 000–50, 000 events per sample was acquired on a BD FASCanto and data analysis was carried out using FlowJo software.

### Measurement of Lactate Dehydrogenase Activity

BMDCs were co-cultured or stimulated with LPS as previously described before the cells were harvested and lysed. Intracellular lactate dehydrogenase activity was measured following manufacturer's instructions. The lactate dehydrogenase activity assay kit was supplied by Sigma-Aldrich (Catlog number: MAK066). This kit reduces NAD to NADH, which is specifically detected as a colorimetric assay.

### Preparation of Extracts for LCMS

BMDCs were co-cultured with *T. gondii* or stimulated with LPS for 24 h before metabolite extraction. The extraction process involved cooling the cells for 10 min on ice to suppress further metabolic changes before the removal of the supernatant. The cells were washed twice with ice cold PBS (Thermo Fisher, UK) and an extraction mixture of cold methanol (VWR Chemicals, Leicestershire, UK), ddH_2_0 and chloroform (VWR Chemicals) in a 60: 20: 60 ratio was added. The cells were scraped thoroughly, and the extracts were shaken at 1,400 rpm for 60 min at 4°C in a thermomixer. After centrifugation at 12,000 × g for 15 min at 4°C, the supernatant from these samples were then transferred to LCMS vials (Sigma-Aldrich/ Merck, Germany) and stored at −80°C.

LCMS was carried out by Glasgow Polyomics, University of Glasgow, UK. Separation was performed using a zwitterionic ZIC pHILIC column (150 × 4.6 mm; 5 uM, Merck) on a Dionex Ultimate 3000 RSLC system (Thermo Fisher) with an injection volume of 10 ul and a flow rate of 0.3 ml/ min. The column was eluted on a gradient of mobile phase A, 20 mM ammonium carbonate pH 9.2 and mobile phase B, acetonitrile (ACN). Mass detection was carried out using an Orbitrap QExactive mass spectrometer (Thermo Fisher) operated in polarity switching mode.

### Data Processing

Raw data files of metabolite standard solutions were processed at the University of Strathclyde using ToxID 2.1 (Thermo Fisher Scientific Inc., Hemel Hempstead, UK) with ± 3 ppm (parts per million) mass accuracy of both ESI positive and negative modes. After checking the appearance of the ion chromatograms with respect to peak shape, the standards were used to calibrate IDEOM v19. Raw files of the sample metabolites from the LCMS were processed by converting the data files to a universally accepted mxXML open file format using msConvert (ProteoWizard). Chromatograms were extracted using a detection algorithm from XCMS and stored in PeakMLfiles before aligning replicate peaks and combining them using mzMatch.R. A CSV file was generated after noise filtering and gap filling (quality control), which was then imported into IDEOM v19 for metabolite identification (Creek et al., 2012). This was based on accurate mass (± 3 ppm) and matching the observed retention time to a database of predicted retention times, based on physicochemical properties of metabolites calculated from their chemical structure. Chromatograms for individual metabolites were examined manually to check peak shape. All metabolites assigned an arbitrary confidence level ≤ 6 by IDEOM, representing a predicted retention time match of <35% (± 21 s), were rejected. Lipids and peptides were also excluded from the putatively identified metabolites in IDEOM.

### Confirmation of Metabolite Identity

The identity of the metabolites was confirmed by accurate mass and matching the sample retention time to that of an authentic standard (± 0.3 min). The confirmed metabolites correspond to the metabolic standards initiative (MSI) level 1 whilst metabolites putatively identified by accurate mass and predicted retention time correspond to MSI level 2.

### RNA-Sequencing

BMDCs activated with LPS or co-cultured with *T. gondii* were harvested after 6 h and the mRNA was extracted from the cell pellets using the RNeasy mini kit (Qiagen) with QIAshredder column (Qiagen, Manchester, UK). An agilent 2100 bio analyser was used to assess the quality of the RNA extracted (Agilent, Cheshire, UK). RNA samples with a concentration >20 ng/μl and RIN >8 was sent to be processed for RNA-seq using the Illuminia sequencing technology at Eurofins GATC Biotech, Konstanz, Germany. Bowtie was used to generate the reference alignments transcriptome alignments to align the RNA-seq reads to the reference transcriptome (mouse). Potential exon-exon splice junctions of the initial alignment were identified by Tophat. Cufflinks (part of CummeRbund software) was then used to identify and quantify the transcripts from a pre-processed RNA-seq alignment assembly. After this, Cuffmerge merges the pieces of the transcripts into full length transcripts and annotated the transcripts. Finally, merged transcripts from two (or more) samples were compared using Cuffdiff to determine the differential expression at transcript level. This includes giving a measure of significance (Benjamini-Hochberg correction) between the samples using fragment per kilobase per million mapped reads (FPKM) for each transcript. For interpretation purposes, each transcript is shown as Log_2_ (fold change) compared to the control and illustrated in a heat map. The quality of the data obtained is outlined in the Supplementary data.

### Mitochondrial Staining

BMDCs were plated at 1 × 10^5^ into a Lab-tek chambered 1.0 borosilicate coverglass system (Catalog no: 155411; Thermo Scientific). Briefly, the cells were washed with PBS before the addition of Tetramethylrhodamine, Methyl Ester, Perchlorate (TMRM) and Mitotracker Green (Thermo fisher). All dyes were added at a final concentration of 100 nM each for 45 min in complete phenol red free RPMI. Cells were washed with PBS to remove extracellular Mitotracker Green, but TMRM was retained in the imaging PBS (as it is freely permeable and equilibrates across membranes in a Nernstian-manner). The cells were placed in a 37°C stage-top chamber (OKO Labs H301-mini) for imaging. BMDCs were imaged on a Nikon Eclipse Ti inverted epifluorescence microscope with a 100x 1.3 NA oil immersion objective lens plus a 1.5x internal microscope adapter lens (Nikon) and Flash 4.0 CMOS camera (Hamamatsu). The samples were imaged with an excitation light of 470nm (Mitotracker Green) and 550 nm (TMRM) (pE4000 LED light source, CoolLED). Camera recording and excitation light controlled by Win Fluor v3.9.1 software (John Dempster, University of Strathclyde). Image analysis was performed using FIJI (Schindelin et al., 2012). All MitoTracker Green and TMRM images were set to the same intensity scale and then converted from 16- to 8-bit to allow application of a mean intensity threshold. Regions of interest were drawn manually around each cell periphery and the threshold set such that only the mitochondria were selected, equally for each pair of images. The “Multi Measure” function of FIJI was then used to measure the mean fluorescence intensity within each cell for both MitoTracker Green and TMRM and the ratio of (mean TMRM)/(mean MitoTracker Green) taken as a measure of relative mitochondrial membrane potential (membrane-potential-dependent fluorophore/membrane-potential-independent fluorophore).

### Cytokine Bead Array (CBA)

Concentrations of different cytokines were determined in the supernatant of naïve BMDCs, LPS stimulated BMDCs and BMDCs co-cultured with *T. gondii* using the Legendplex™ Mouse Inflammation Panel (Cat no; 740150; Biolegend, UK). The assay was carried out as per the manufacturer's instructions.

### Data Analysis

Prior to Principal component analysis (PCA) and Orthogonal partial least squares discriminant analysis (OPLS-DA), the data were mean-centered and Pareto (Par) scaled. PCA, OPLS, and VIP (Variable importance in the projection) plots were performed using SIMCA-P13 (Umetrics, Sweden). For metabolomics data, a non-parametric one-tailed Mann Whitney test was used for all individual putatively identified metabolites. *p* < 0.05 was considered significant. Graph Pad prism 7 was used for plotting the graphs and heat maps. Heat-maps show the fold change in each metabolite compared to the control. All functional data was performed by a one-way ANOVA with either a Bonferroni or Dunnett post-test unless otherwise stated. Throughout this thesis, ^*^p < 0.05, ^**^p < 0.01, and ^***^p < 0.001.

## Results

### *T. gondii* Actively Invades BMDCs

**Figure 1:**
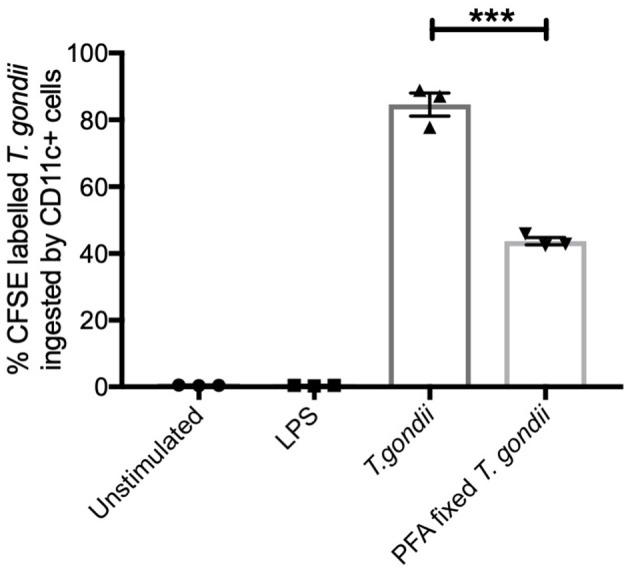
*T. gondii* actively invades BMDCs. Bone marrow derived DCs were co-cultured with CFSE labeled *T. gondii* tachyzoites (MOI 1:1). After 6 h, cells were analyzed by flow cytometry to determine CFSE parasite uptake. Results are representative of three independent experiments and show mean ± SEM (*n* = 3). A one-way ANOVA was performed with a Bonferroni post-test to determine significance (*P* < 0.01) where ***p < 0.001.

CFSE labeled *T. gondii* parasites (MOI 1:1) were co-cultured with BMDCs to establish the extent these parasites were internalized. Flow cytometry was used to identify CFSE labeled *T. gondii* in CD11c+ cells. Paraformaldehyde fixed parasites were used as a comparator to determine the potential of BMDCs to internalize dead parasites. CFSE labeled live *T. gondii* were found in approximately 80% of CD11c^+^ cells while PFA fixed *T. gondii* were found in significantly fewer BMDCs (approximately 40%) (Figure 1).

### *T. gondii* Augments CD40, CD80 and CD86 Transcript and Protein Levels, but Down Regulates MHC Transcripts

**Figure 2:**
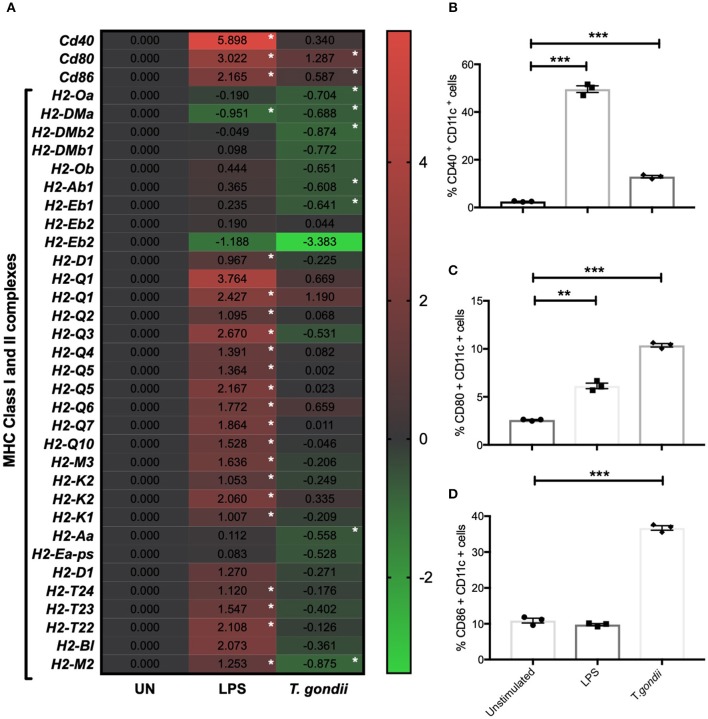
*T. gondii* alters the cellular activation transcript and marker expression of BMDCs. Bone marrow derived DCs were co-cultured with *T. gondii* tachyzoites (MOI 1:1) or stimulated with LPS as indicated for 6 or 24 h. After 6 h, **(A)** mRNA was subsequently extracted and then quantified using RNA-seq. Data shows the log_2_ (fold change) as normalized to unstimulated controls. Red indicates an increase in mRNA expression, green indicates a decrease in transcript expression and gray shows no changes from the control cells. In addition, cells were analyzed by flow cytometry after 24 h to determine the proportion of CD11c+ that cells expressed the activation markers **(B)** CD40, **(C)** CD80, or **(D)** CD86. Results are representative of one experiment for transcriptomics and three independent experiments for flow cytometry and show mean ± SEM. Statistical analysis was performed **(A)** using Cuffdiff (Eurofin) to determine the differential expression levels at the transcript level including a measure of significance between samples. **(B–D)** one-way ANOVA with Bonferroni post-test was performed to determine significance (*P* < 0.05) where *p < 0.05, **p < 0.001 and ***p < 0.0001 are significant compared to unstimulated.

CD80 and CD86 work in concert with MHCI and MHCII to activate T cells. The activation profiles of BMDCs co-cultured with *T. gondii* or stimulated with LPS were determined by flow cytometry and transcriptomics. In comparison to naïve BMDCs, those co-cultured with *T. gondii* or stimulated with LPS had significantly increased CD40 and CD80 protein levels (Figures 2B,C). In contrast CD86 was only seen to be upregulated in BMDCs co-cultured with *T. gondii* (Figure 2C). Many of these changes also were evident in the transcriptomic data including *cd80* and *cd86* mRNA transcripts which were upregulated in BMDCs co-cultured with *T. gondii*. Similarly, transcripts for *cd40, cd80, and cd86* were significantly upregulated in LPS stimulated BMDCs. (Figure 2A). A number of transcripts for MHC class I and II molecules were significantly downregulated in BMDCs co-cultured with *T. gondii*. In contrast, transcripts for MHC class I and II molecules were generally significantly upregulated in BMDCs stimulated with LPS compared with control cells (Figure 2A).

### *T. gondii* Influences the Cytokine Profile of BMDCs at Both the mRNA and Protein Level

**Figure 3:**
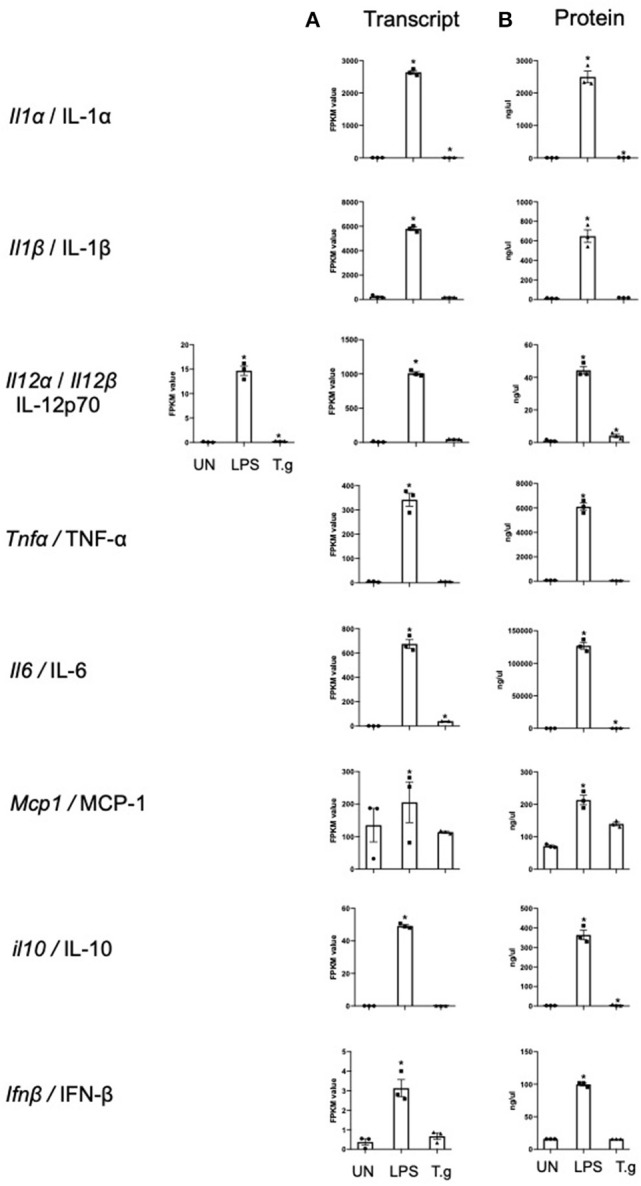
mRNA and protein levels of cytokines expressed by BMDCs when co-cultured with *T. gondii* or stimulated with LPS. Bone marrow derived DCs were either co-cultured with *T. gondii* or stimulated with LPS as indicated for 6 or 24 h. After 6 h, **(A)** mRNA was subsequently extracted and then quantified using RNA-seq. Data shows the FPKM values. Red indicates an increase in mRNA expression, green indicates a decrease in transcript expression and gray shows no changes from the control cells. **(B)** A cytokine bead array was performed on the supernatant to obtain cytokine protein levels. Results are representative of one experiment for transcriptomics and three independent experiments for flow cytometry and show mean ± SEM. Statistical analysis was performed **(A)** using Cuffdiff (Eurofin) to determine the differential expression levels at the transcript level including a measure of significance between samples. **(B)** One-way ANOVA with Dunnett post-test. Significant differences are *p* < 0.05 where * is compared to unstimulated.

Cytokine production by BMDCs co-cultured with *T. gondii* or stimulated with LPS were measured in culture supernatants by cytometric bead array (CBA) and compared with the transcriptomic data to validate the RNA sequencing process. The cytokine protein levels of IL-1α, IL-12p70, IL-6, and IL-10 were significantly increased in *T. gondii* co-cultured BMDCs. This was largely consistent with the transcriptomic data which found significant upregulation of *Il1a, Il12a*, and *Il6* transcripts. Similarly, LPS significantly increased the mRNA and protein levels compared to naïve BMDCs, for of all cytokines measured (Figure 3).

### Dendritic Cells Co-cultured With *T. gondii* or Stimulated With LPS Undergo Distinct but Overlapping Transcript and Metabolic Changes

**Figure 4:**
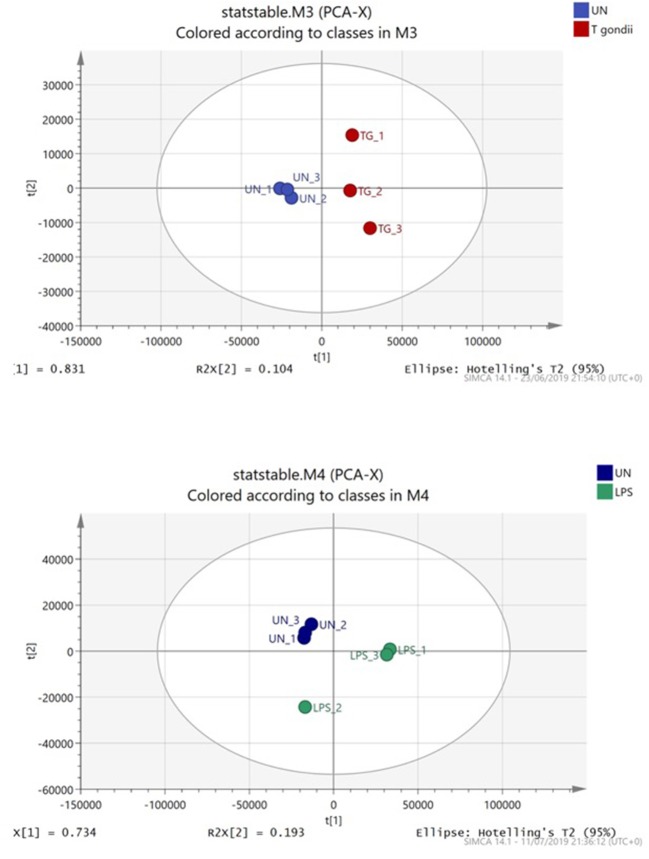
Principal component analysis of BMDCs co-cultured with *T. gondii* or activated with LPS. Bone marrow derived DCs were co-cultured with *T. gondii* or stimulated with LPS for 24 h. After this time, the metabolites of the DCs were extracted and run via Liquid chromatography mass spectroscopy (LCMS). The data was then analyzed by SIMCA and a Principal component analysis plot was generated. Key: Blue, unstimulated; Green, LPS and Red, *T. gondii*.

Principal component analysis (PCA) demonstrated distinct separation between *T. gondii* infected and naïve BMDCs (Figure 4). Furthermore, Orthogonal partial least squares discriminant analysis (OPLS-DA) was used to generate a ranked table of variable importance in projection (VIP) table for metabolites and transcripts (Supplementary Tables 1–4). The transcripts were aligned to a *Mus musculus* reference gene ensuring there is no *T. gondii* specific transcripts and all raw signal values as well as fold- change values for this entire data set are publicly available (Supplementary Table 5).

As expected, most transcripts scoring high in the pair wise OPLS-DA analyses comparisons between control cells and *T. gondii* co-cultured or LPS stimulated cells were immune related or involved with signaling molecules/transcription factors. Interestingly, many transcripts associated with energy metabolism including glycolysis, TCA cycle and OXPHOS as well as arginine metabolism were also ranked within the initial 100 transcripts of interest (Supplementary Tables 1, 2). Similarly, multiple metabolites from these pathways were highlighted within the first 50 metabolites of the metabolome VIP tables and heatmaps (Supplementary Tables 3, 4 and Supplementary Figure 1). From both VIP tables, it is clear that *T. gondii* induces metabolic changes in BMDCs.

Within this paper, only transcript and metabolic changes associated with live *T. gondii* infection will be discussed in great detail (Supplementary Figure 1). A limitation of investigating the metabolic profile of BMDCs infected with live parasites is distinguishing whether deviations of the metabolites described herein are associated with changes in BMDC function or reflect metabolites of the parasite. In an attempt to understand the host contribution to the observed metabolic changes in the absence of live parasites, paraformaldehyde (PFA) fixed *T. gondii* or *Toxoplasma* lysate antigen (TLA) were also used to stimulate BMDCs. Results found either a marked reduction or only relatively minor changes in cultures incubated with PFA fixed parasites or TLA compared unstimulated BMDCs. This implies that activation of parasite specific TLR receptors alone is insufficient to modify the metabolism of BMDCs and that an active infection is required (Supplementary Figure 1.7). Consequently, we used both metabolomic data and transcriptomic analysis in concert to gain insight into the likely contribution of the host metabolism during *T. gondii* infection.

### *T. gondii* Infection Induces Aerobic Glycolysis of BMDCs

**Figure 5:**
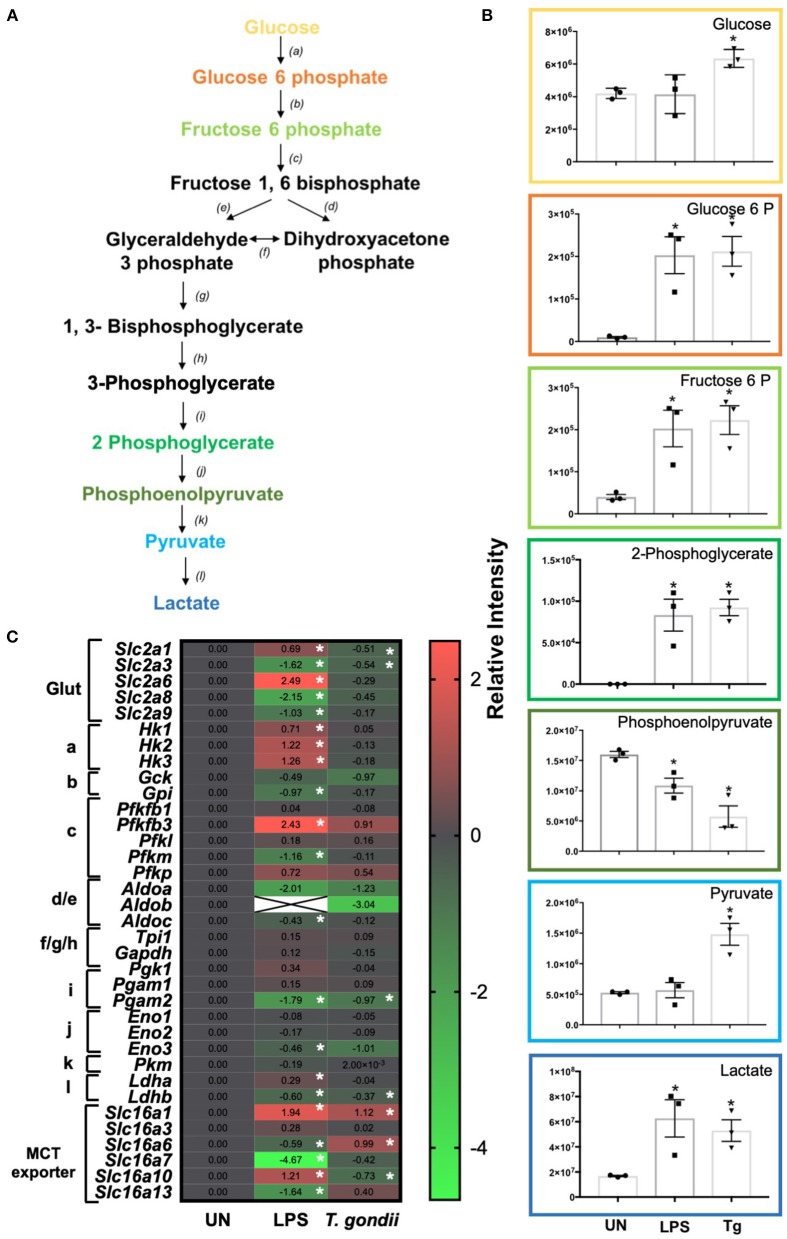
Glycolysis differences in *T. gondii* co-cultured or LPS stimulated BMDCs. Bone marrow derived DCs were co-cultured with *T. gondii* or stimulated with LPS as indicated for 6 or 24 h. After this time, the metabolites of the BMDCs were **(B)** extracted and analyzed by Liquid Chromatography Mass Spectroscopy (LCMS) or **(C)** mRNA was extracted and then quantified using RNA-seq. Data shows the log_2_ (fold change) as normalized to unstimulated controls. Red indicates an increase in transcript expression, green indicates a decrease in transcript expression and gray shows no changes from the control cells. For clarity, the diagram shown in **(A)** focuses on relevant metabolites (shown in the graphs) and enzymes [depicted **(a)** through to **(l)**] for glycolysis only where **(a)** hexokinase; **(b)** phosphoglucose isomerase; **(c)** phosphofructokinases; **(d,e)** aldolase; **(f)** triose phosphate isomerase; **(g)** glyceraldehyde 3 phosphate dehydrogenase; **(h)** phosphoglycerate kinase; **(i)** phosphoglycerate mutase **(j)** enolase; **(k)** pyruvate kinase and **(l)** lactate dehydrogenase. Statistical analysis was either performed using **(B)** a non-parametric Mann Whitney test, **(C)** Cuffdiff (Eurofin) to determine the differential expression levels at the transcript level including a measure of significance between samples. Significant differences are *p* < 0.05 where * is compared to unstimulated. Results are representative of three independent runs.

Glycolysis is the first step in the breakdown of glucose to produce the high-energy molecules, ATP and NADH. Within this pathway, glucose is metabolized into pyruvate (under aerobic conditions) or lactate (in an anaerobic environment) (Figure 5A).

Distinct differences in glycolytic transcripts and metabolites were observed in BMDCs following co-culture with *T. gondii* or following LPS stimulation for 24 h. LCMS analysis detected increased levels of the glycolytic metabolites, glucose, glucose 6 phosphate, fructose 6 phosphate, 2 phosphoglycerate, pyruvate and lactate in BMDCs co-cultured with *T. gondii* compared with control BMDCs (Figure 5B). These differences were similar to those observed in LPS-stimulated BMDCs. Transcript levels generally did not reflect this increase in glycolysis as BMDCs co-cultured with *T. gondii* had decreased levels of key transcripts encoding glycolytic enzymes phosphoglycerate mutase (*pgam2*) and enolase (*eno3*). Transcription of genes for the glucose transporters GLUT1 (slc2a1) and GLUT3 (slc2a3) was also reduced but there were modest increases in monocarboxylate transporters MCT1 (*slc16a1*) and MCT6 (*slc16a6*) (Figure 5C). MCT1 is a proton dependent transporter for lactate, pyruvate and ketone bodies (Fisel et al., 2018). MCT6 has the similar substrate specificity *in vitro* but its function is unknown. BMDCs stimulated with LPS had significantly increased levels of glucose transporters GLUT1, GLUT6 (*slc2a1 and slc2a6)*, hexokinases (*hk1, hk2, hk3*), 6 phosphofructokinase b3 (*pfkfb3*), lactate dehydrogenase a (*ldha*) and monocarboxylate transporters MCT1 (slc16a1) and MCT10 (slc16a10). There were significantly decreased levels of glucose transporters GLUT3, GLUT8 and GLUT9 (*slc2a3, slc2a8, slc2a9*) glycolytic enzymes glucose 6 phosphate isomerase (*gpi*), 6 phosphofructokinase m (*pfkm*), aldolase c (*aldoc*), phosphoglycerate mutase (*pgam*) and monocarboxylate transporters MCT6, MCT7, and MCT13 (*slc16a6, slc16a7, and slc16a13*) (Figure 5C). MCT10 is required for transport of aromatic amino acids, GLUT9 has been implicated in urate transport but the physiological roles of GLUT6, GLUT8, MCT6, MCT7, and MCT13 have not been determined (Cura and Carruthers, 2012; Fisel et al., 2018). Overall, observed transcripts and metabolites associated with glycolysis trend toward an induction of this pathway upon *T. gondii* co-culture and LPS stimulation.

### *T. gondii* Increases Glucose Uptake and Up-Regulates LDH Activity in BMDCs

**Figure 6:**
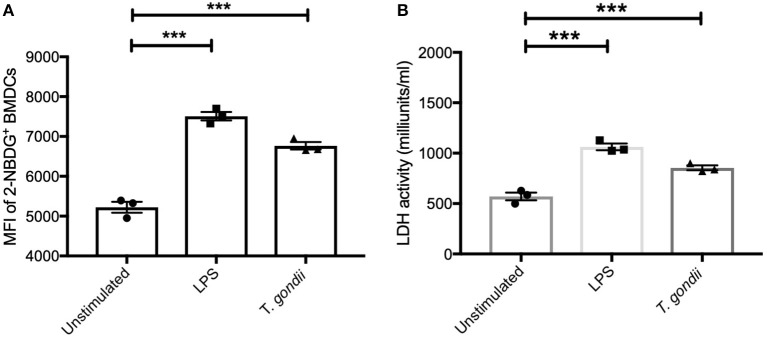
Glucose uptake and Lactate dehydrogenase activity were increased in BMDCs exposed to *T. gondii*. Bone marrow derived DCs were stimulated with LPS or co-cultured with *T. gondii* (1:1 ratio). After 24 h **(A)** fluorescent 2-NBDG (50 μM) was added for 120 min. The mean fluorescent intensity (MFI) of 2-NBDG uptake in BMDCs was then quantified using flow cytometry. **(B)** Supernatant from homogenized cells was used to calculated the LDH activity based on LDH reducing NAD to NADH. Results are representative of three independent experiments (*n* = 3) and show the mean ± SEM. Statistical analysis performed using 1-way ANOVA with Bonferroni post-test. Significant differences were identified where ***p < 0.001.

To further confirm the observed increase in glycolysis highlighted in the transcriptomics and metabolomic data, the ability of *T. gondii* to alter BMDC uptake of 2-NBDG (as a proxy for glucose) and influence lactate dehydrogenase (LDH) activity was used. Co-culture of BMDCs with *T. gondii* significantly increased glucose uptake and elevated LDH activity compared with control BMDCs. An increase in glucose uptake and up-regulation in LDH activity was also observed in BMDCs stimulated with LPS (Figures 6A,B). These findings confirm that upon *T. gondii* infection, BMDCs increase their capability to take up glucose favoring glycolysis.

### *T. gondii* Infection Alters the TCA Cycle in BMDCs

**Figure 7:**
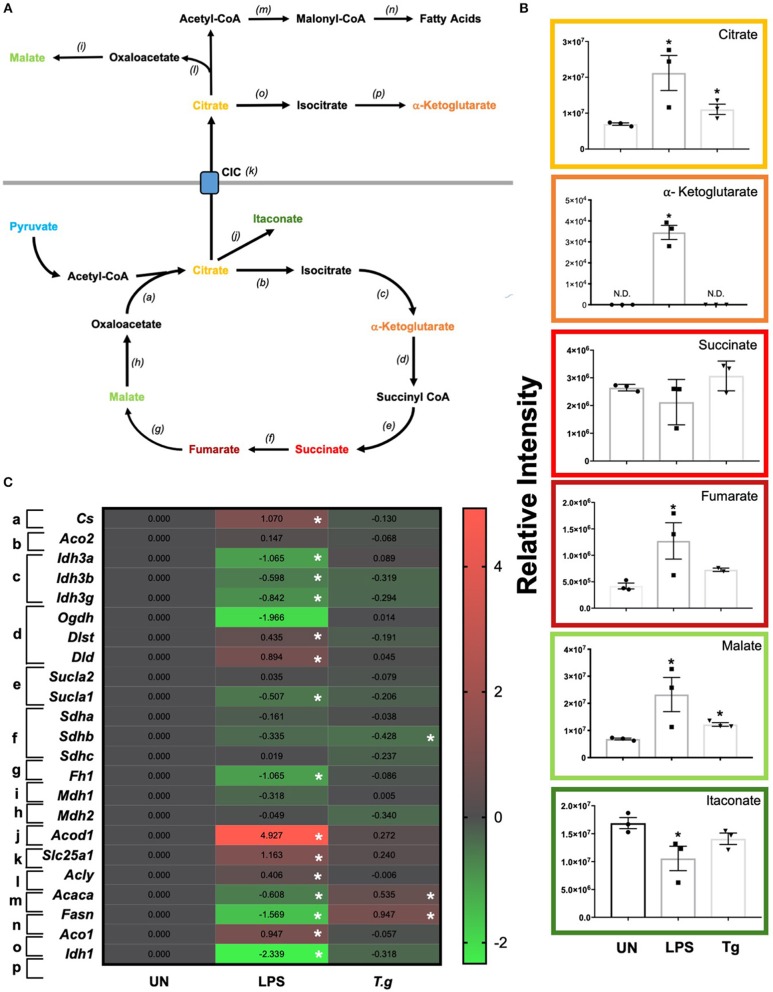
TCA changes in *T. gondii* co-cultured or LPS stimulated BMDCs. Bone marrow derived DCs were co-cultured with *T. gondii* or stimulated with LPS as indicated for 6 or 24 h. After this time, the metabolites of the BMDCs were **(B)** extracted and analyzed by Liquid Chromatography Mass Spectroscopy (LCMS) or **(C)** mRNA was extracted and then quantified using RNA-seq. Data shows the log_2_(fold change) as normalized to unstimulated controls. Red indicates an increase in transcript expression, green indicates a decrease in transcript expression and gray shows no changes from the control cells. For clarity, the diagram shown in **(A)** focuses on relevant metabolites (shown in the graphs) and enzymes [depicted **(a)** through to **(j)**] for the TCA cycle only. **(a)** citrate synthase; **(b)** aconitase 2; **(c)** isocitrate dehydrogenase 3; **(d)** alpha-ketoglutarate dehydrogenase; **(e)** succinyl-CoA synthetase; **(f)** succinate dehydrogenase; **(g)** fumarase; **(h,i)** malate dehydrogenase; **(j)** cis-aconitate decarboxylase; **(k)** mitochondrial citrate carrier; **(l)** ATP citrate lyase; **(m)** acetyl CoA carboxylase; **(n)** fatty acid synthase; **(o)** aconitase 1 and **(p)** isocitrate dehydrogenase 1. Statistical analysis was either performed using **(B)** a non-parametric Mann Whitney test, **(C)** Cuffdiff (Eurofin) to determine the differential expression levels at the transcript level including a measure of significance between samples. Significant differences are *p* < 0.05 where * is compared to unstimulated. Results are representative of three independent runs (*n* = 3).

Once metabolized from pyruvate (the end product of glycolysis), acetyl-CoA can enter the TCA cycle. The key reaction of the cycle is the reduction of NAD+ into NADH, which can then be introduced into the electron transport chain (i.e., OXPHOS) to generate ATP (Figure 7A). LCMS analysis from co-culturing *T. gondii* with BMDCs for 24 h demonstrated increased levels of citrate and malate in *T. gondii* co-cultured BMDCs (Figure 7B). A decrease in the majority of mRNA transcripts in these cultures could be observed although only succinate dehydrogenase B (*sdhb*) was statistically significant in itself (Figure 7C). Similarly, many TCA intermediates were up-regulated in LPS stimulated BMDCs including citrate, alpha-ketoglutarate, fumarate and malate. Itaconate was observed to be significantly down-regulated compared to naïve cells (Figure 7B). Significant down-regulation of transcripts for isocitrate dehydrogenase A, B and G (*idh3g, idh3b, idha*), alpha ketoglutarate dehydrogenase (*ogdh*), succinate-coA ligase (*suclg1*) and fumarate hydratase (*fh*) were evident in LPS activated BMDCs in comparison to unstimulated BMDCs. Significant upregulation of dihydrolipoamide S-succinyltransferase (*dlst*) were observed in LPS stimulated BMDCs (Figure 7C).

Transcripts for citrate synthase (*cs*) were upregulated in LPS stimulated, but not *T. gondii* infected BMDCs. Similarly, the citrate carrier (slc25a1) that transports citrate from the mitochondria to the cytoplasm was markedly upregulated in LPS stimulated BMDCs, but only modestly raised in *T. gondii* infected BMDCs. Examination of potential routes for citrate usage in the cytoplasm of LPS stimulated cells revealed an increase in ATP citrate lyase (*acly*) which is responsible for the conversion of citrate to acetyl coA. However, transcripts for acetyl-CoA carboxylase (*acaca*) and fatty acid synthase (*fasn*) which consecutively convert acetyl coA to fatty acid were downregulated in these cells. In contrast in *T. gondii* infected cells no change in ATP citrate lyase (*acly*) transcripts were noticed, but an increase in acetyl-CoA carboxylase (*acaca*) and fatty acid synthase (*fasn*) were observed.

Generally, from our multi-omics data-set, we observed a decrease in the majority of TCA transcripts, but an increase in many metabolites associated with the TCA cycle. This is in agreement with current metabolomic studies in LPS stimulated macrophages and dendritic cells that show blockage of specific TCA cycle enzymes leading to the increase of certain intermediates. It has been shown that these metabolites have an immunoregulatory role within host cell cells.

### *T. gondii* Effects on the Electron Transport Chain and Mitochondrial Membrane Potential

**Figure 8:**
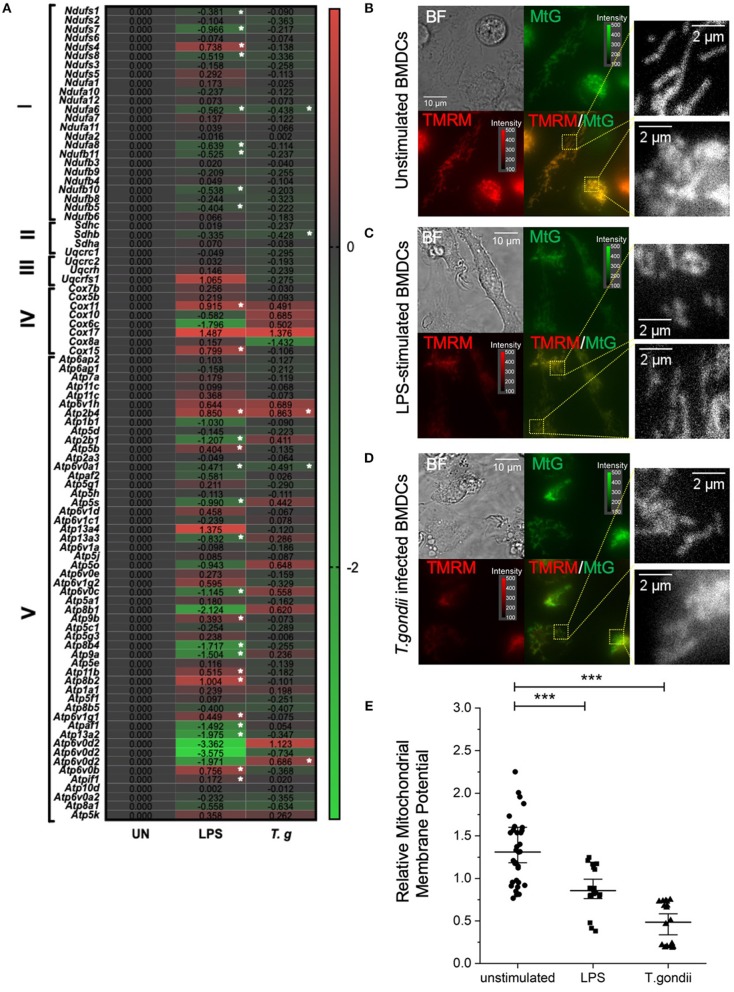
*T. gondii* infection and LPS activation decreases mitochondria membrane potential within BMDCs. Bone marrow derived DCs were co-cultured with *T. gondii* (1:1 ratio) or activated with LPS. **(A)** After 6 h, mRNA was subsequently extracted and then quantified using RNA-seq. Data shows the Log_2_(fold change) as normalized to unstimulated controls. Red indicates an increase in transcript expression, green indicates a decrease in transcript expression and gray shows no change from control cells. For Clarity, relevant gene abbreviates are shown in the diagram where Complex I is NADH dehydrogenase (*ndufs1-bdufb*6), Complex II is Succinate dehydrogenase (*sdhc-sdha*), Complex III is Cytochrome bc1 complex (*uqcrc1-uqcrfs1*), Complex IV is Cytochrome C oxidase (*cox7b–cox15*) and Complex V is ATP synthase (*Atp6ap2–atp5k*). Statistical analysis was performed using Cuffdiff (Eurofin) to determine the differential expression levels at the transcript including a measure of significance between samples. **(B–D)** After 24 h, the cells were stained with 100 nM of Mitotracker Green and 100 nM TMRM for 30 min to determine mitochondrial morphology. The images show separate images for Bright field (BF), Mitotracker green (MtG), TMRM and then MtG and TMRM merged (TMRM/MTG). Examples of areas used for quantification of relative mitochondrial potential are shown zoomed in yellow dashed boxes. Images are representative of at least 3 independent runs per group, with excitation light intensity and image intensity kept constant throughout. **(E)** Relative mitochondrial membrane potential for control BMDCs, BMDCs stimulated with LPS or BMDCs infected with *T. gondii* (determined as described in the material and methods section). Statistical analysis performed using non-parametric Kruskal-Wallace ANOVA with Dunn's *post-hoc* test (OriginPro), with ***p < 0.001; individual cell values overlaid with median ± 95% confidence interval.

Due to the direct link between the TCA cycle and electron transport chain, the effect of *T. gondii* infection on transcripts encoding components of the electron transport chain (ETC) was assessed. Down regulation of components of the ETC was observed in *T. gondii* infected and LPS stimulated BMDCs. Generally, complex I (NADH dehydrogenase), complex II (succinate dehydrogenase) and complex 3 (cytochrome bc1 complex) were unaffected or downregulated (of note, *ndufa6 and sdhb* were significantly (p <0.05) down-regulated). Previous literature shows that a downregulation of the electron transport chain is expected upon BMDC activation, as limiting ATP production for energy allows TCA metabolites to be used in other biosynthetic pathways e.g., fatty acid biosynthesis. However, interestingly, transcripts encoding subunits of complex IV and V (ATP synthase) were generally found to be upregulated in BMDCs following exposure to *T. gondii*. Statistically significantly upregulated subunits were *atp6v0b* and *atp2b4*. This is a relatively new concept with studies demonstrating that subunits IV and V of the ETC have roles other than energy generation including heme A biosynthesis (*cox11*), transport of different ions and maintenance of calcium homeostasis (*atp2b4*), mediating acidification (*atp6v0b*) (Figure 8A).

Moreover, important differences in the mitochondrial membrane potential (an indicator of oxidative phosphorylation) between naïve and *T. gondii-*infected BMDCs were observed using epifluorescence microscopy. After 24 h of co-culture between BMDCs and *T. gondii*, Mitotracker Green was used to stain mitochondria in combination with TMRM to visualize mitochondrial membrane potential. TMRM accumulates in active mitochondria with an intact membrane potential. A color change from red/orange to yellow/green when Mitotracker green and TMRM signals were merged demonstrates that *T. gondii* and LPS reduce mitochondrial membrane potential (Figures 8B–D). Furthermore, significant downregulation in the relative mitochondrial membrane potential (TMRM/MTG) mean per cell of *T. gondii* infected and LPS stimulated BMDCs could be observed compared to naïve BMDCs (Figure 8E). Overall, this indicates a decrease in oxidative phosphorylation in *T. gondii* infected and LPS stimulated BMDCs.

### *T. gondii* Infected BMDCs Direct Arginine Metabolism to Polyamines and Proline

**Figure 9:**
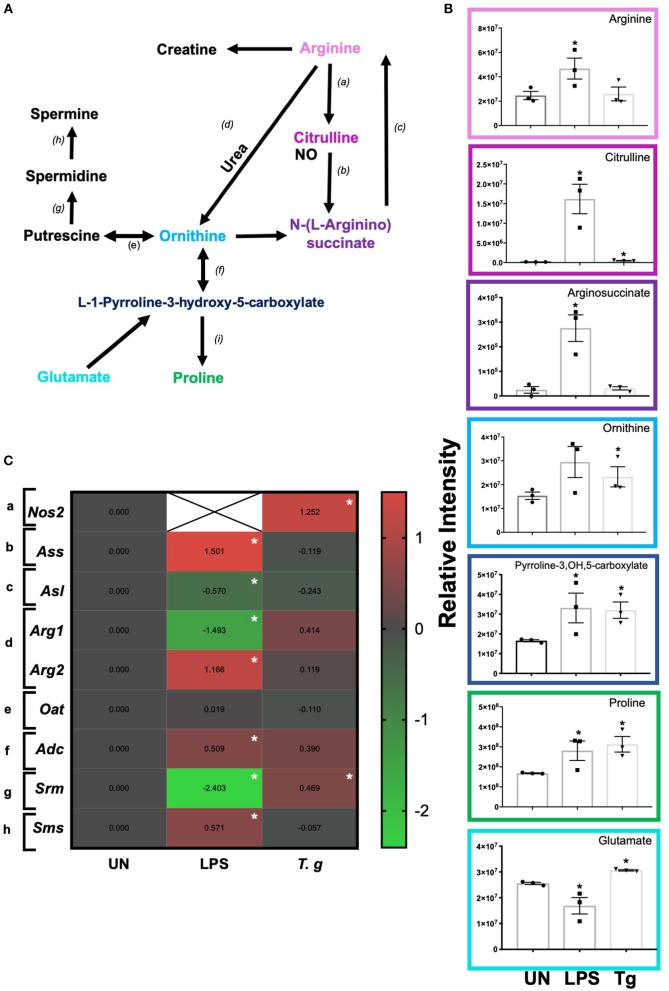
Arginine metabolism changes in BMDCs co-cultured with *T. gondii* or stimulated with LPS. Bone marrow derived DCs were co-cultured with *T. gondii* or stimulated with LPS as indicated for 6 or 24 h. After this time, the metabolites of the BMDCs were **(B)** extracted and analyzed by Liquid Chromatography Mass Spectroscopy (LCMS) or **(C)** mRNA was extracted and then quantified using RNA-seq. Data shows the log_2_(fold change) as normalized to unstimulated controls. Red indicates an increase in transcript expression, green indicates a decrease in transcript expression and gray shows no changes from the control cells. For clarity, the diagram shown in **(A)** focuses on relevant metabolites (shown in the graphs) and enzymes [depicted **(a)** through to **(h)**] for arginine metabolism only: **(a)** NOS; **(b)** argininosuccinate synthase; **(c)** argininosuccinate lyase; **(d)** arginase; **(e)** ornithine decarboxylase; **(f)** ornithine aminotransferase; **(g)** spermidine synthase; **(h)** spermine synthase; and **(i)** △1-pyrroline- 5-carboxylate reductase. Statistical analysis was performed using **(B)** non-parametric Mann Whitney test, **(C)** Cuffdiff (Eurofin) to determine the differential expression levels at the transcript including a measure of significance between samples. Significant differences are *p* < 0.05 where * is compared to unstimulated. Results are representative of three independent runs (*n* = 3).

Independent of energy metabolism in *T. gondii* infected BMDCs, RNA sequencing and LCMS analysis detected a number of transcripts and metabolites associated with arginine metabolism in *T. gondii* co-cultured BMDCs (Figure 9A). The metabolism of arginine is associated with increased production of toxic mediators deleterious to many pathogens. Notably, in *T. gondii* infected BMDC cultures co-cultured for either 6 h (RNA) or 24 h (metabolites), arginine levels were similar to control BMDCs and only a small increase in citrulline levels was evident. However, significant increases in L-ornithine, L-proline, L-1-pyrroline-3-hydroxy-5-carboxylate and L-glutamate were observed in *T. gondii* co-cultured BMDCs. This differed from LPS stimulation which induced significantly increased levels of citrulline, N-(omega)-hydroxyarginine and arginosuccinate as well as L-proline and L-1-pyrroline-3-hydroxy-5-carboxylate (Figure 9B). Transcripts for a number of enzymes involved in arginine conversion to proline and glutamate were also seen to be collectively augmented, but none other than spermidine synthase (*srm)* were individually significantly increased in *T. gondii* infected BMDC cultures (Figure 9C).

**Figure 10:**
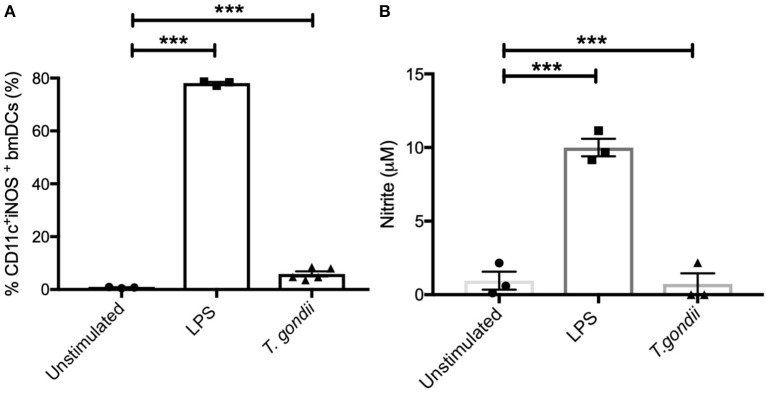
Intracellular iNOS expression and NO production is not altered in BMDCs infected with *T. gondii*. Bone marrow derived DCs were co-cultured with *T. gondii* (1:1 ratio) or stimulated with LPS. After 24 h, **(A)** CD11c+ cells were analyzed for iNOS expression by flow cytometry. **(B)** supernatants were used to measure NO by Griess assay. Results are representative of three independent experiments and show the mean ± SEM. Statistical analysis performed using 1-way ANOVA with Bonferroni post-test. Significant differences were identified where ***p < 0.001.

**Figure 11:**
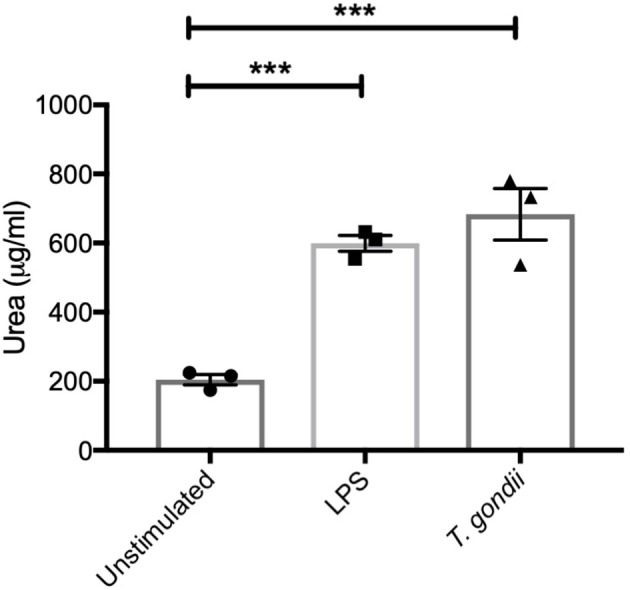
BMDCs up-regulate Arginase-1 activity in response to *T. gondii*. Bone marrow derived DCs were co-cultured with *T. gondii* (1:1 ratio) or stimulated with LPS. After 24 h, Urea (μg/ml) was measured from homogenized cells. This is indicator of Arginase-1 activity. Results are representative of three independent experiments (*n* = 3) and show the mean ± SEM. Statistical analysis performed using 1-way ANOVA with Bonferroni post-test. Significant differences were identified where ***p < 0.001.

In keeping with the omics data, intracellular iNOS was detected in 80% of CD11c+ BMDCs stimulated with LPS, but in <10% of cells from *T. gondii* infected cultures. Furthermore, nitrite levels in LPS stimulated, but not *T. gondii*-infected BMDCs were significantly increased (Figures 10A,B). Arginase enzyme activity was upregulated in BMDCs cultures infected with *T. gondii* or stimulated with LPS. Collectively, these observations support that arginine is primarily converted to ornithine and polyamines in *T. gondii* infected cells (Figure 11). In contrast, LPS stimulation primarily induces arginine conversion to citrulline and recycling through N-(L-arginosuccinate) with accompanying NO production. By combining metabolomic, transcriptomic data with conventional functional studies reveals that *T. gondii* is able to divert arginine favorably toward ornithine and proline via arginase.

## Discussion

Previous studies using microarray analyses have examined global changes in gene expression for human foreskin fibroblasts, macrophages and DCs in response to *T. gondii* (Blader et al., 2001; Chaussabel et al., 2003). These studies were conducted before the development of RNASeq (which allows almost complete coverage of the transcriptome) and non-targeted metabolomic analyses LCMS techniques. The current studies were initiated to make use of both these modern techniques in concert with conventional functional studies to validate the findings. We elected to use murine cells to complement and enrich the datasets available and to allow extrapolation to the many murine studies already published.

Recent studies have described how macrophages and to some extent BMDCs undergo metabolic changes when exposed to environmental stimuli. Generally, LPS stimulation culminates in a shift from oxidative phosphorylation to aerobic glycolysis, whereas augmented oxidative phosphorylation and ATP production occurs in those stimulated with IL-4 (Newsholme et al., 1986; Jantsch et al., 2008; Rubic et al., 2008; Krawczyk et al., 2010; Infantino et al., 2011; Everts et al., 2012; O'Neill and Hardie, 2013; Tannahill et al., 2013; Jha et al., 2015; O'Neill and Pearce, 2016). However, the effect of intracellular pathogens on cellular metabolism is less well-studied and indeed more complex as host cells undergo metabolic changes that have evolved to directly kill pathogens or restrict access to essential nutrients (Denkers, 2010; Pifer and Yarovinsky, 2011). Conversely pathogens have evolved mechanisms to subvert host cell metabolism for their own benefit. As these early events are important for establishment of infection, initiating host immunity and determining the eventual success of infection we have employed a multi-omics approach to inform confirmatory functional assays.

Initial studies determined that using the procedures outlined, a *T. gondii* infection rate of approximately 80% could be achieved in BMDCs with concurrent upregulation of costimulatory molecules (CD40, CD80, and CD86) and their transcripts *cd80 and cd86*. A similar pattern of changes was also observed by BMDCs stimulated with LPS. This is consistent with the literature (Verhasselt et al., 1997; Morgado et al., 2014). In keeping with the literature that demonstrates the ability of *T. gondii* infection to inhibit MHCI and II expression in macrophages, the majority of MHC Class I and II transcripts were down-regulated in *T. gondii* co-cultured BMDCs (Lüder et al., 2001). In contrast, LPS stimulated BMDCs had elevated levels of many transcripts associated with MHC I and II complex. Additionally, *T. gondii* infected BMDCs had congruent elevated expression of both mRNA and protein levels of pro-inflammatory cytokines IL-1α, IL-12, and IL-6 (Nam et al., 2011). These data validate the model of infection and the multi-omics approach where metabolomic, transcriptomic data and functional studies can complement, be related to, and add value to each other.

Principal component analysis and Orthogonal projection to latent structure for discriminant analyses (OPLS-DA) was used to determine the major changes identified through RNA sequencing and non-targeted liquid chromatography mass spectroscopy (LCMS). From the VIP tables generated from our OPLS-DA analysis, metabolites associated with energy metabolism including glycolysis, the TCA cycle and oxidative phosphorylation were determined as major discriminants (i.e., the first 100 transcripts/ metabolites) between control uninfected and *T. gondii* infected cells.

Glycolysis is metabolic pathway that converts glucose into pyruvate. The energy released in this process is used by downstream metabolic processes to form ATP and NADH. From transcriptomic and metabolic analyses, *T. gondii* co-cultured with BMDCs for 24 h demonstrated an increase in glycolysis intermediates (glucose 6 phosphate, fructose 6 phosphate, 2 phosphoglycerate, pyruvate and lactate) when compared with control BMDCs. Our results also found that LPS stimulation of dendritic cells had a similar effect on glycolysis intermediates. In keeping with these results, we noted increased levels of transcripts for some glycolytic enzymes including hk and pfkfb3, the highly active isoform of phosphofructokinase. We noted a reduction in phosphoenolpyruvate in infected cultures, which could be linked to the downregulation of Eno3 or alternatively due to parasite utilization of this resource. Similarly, up-regulation of ACCase and FAS may allow *T. gondii* to take advantage of the increased production of pyruvate and acetyl-CoA from TCA intermediates that is then used to increase glycolytic demand or FA synthesis (in the apicoplast of the parasite). Conversely, the parasite could contribute to the observed increase in pyruvate we report in infected cultures. Thus, *T. gondii* has the opportunity to contribute to metabolites measured and to exploit host cell resources. Again, LPS stimulated BMDCs underwent similar, but more profound changes to their transcripts encoding glycolytic intermediates.

Through functional assays we were able to demonstrate that even though *T. gondii* infected BMDC had reduced transcript expression of the glucose transporter GLUT1 (*slc2a1*), they had increased uptake of 2-NBDG as a surrogate for glucose and had increased levels of LDH activity compared with control BMDCs. This apparent discrepancy could be due to temporal differences in RNA expression and GLUT1 activity or reflect increased efficiency of transport of glucose due to increased utilization, recruitment of intracellular sugar transporters to the plasma membrane or activation of GLUT1 by reduction in ATP levels (Cura and Carruthers, 2012). Increased levels of lactate and LDH activity was accompanied by increased expression of MCT1, a protein responsible for lactate efflux required to prevent intracellular acidification (Fisel et al., 2018). Interestingly, LDH activity also correlates with an increase metabolites (3-(4- hydroxyphenyl) lactate and indolelactate) produced by LDH from products of aromatic amino acid catabolism. Collectively, these results demonstrate that both *T. gondii* infection of BMDCs and LPS stimulation of BMDCs increase glucose uptake, glycolysis and lactate production in cells even under normoxic conditions, a phenomenon sometimes referred to as aerobic glycolysis or the Warburg effect (Warburg et al., 1958; Reviewed by Rodríguez-Prados et al., 2010; Kelly and O'Neill, 2015).

The Warburg effect is not only associated with aerobic glycolysis, but also by a down-regulation of the TCA cycle and this has already been reported in macrophages stimulated with LPS (Jha et al., 2015). The majority of mRNA transcripts associated with the TCA cycle were down-regulated in LPS activated and *T. gondii* infected BMDCs compared to naïve BMDCs. As a predicted physiological consequence of such circumstances we also detected increased levels of many TCA intermediates in *T. gondii* infected BMDCs and LPS stimulated BMDCs including citrate and malate. In the literature, a build-up of specific TCA intermediates and reduction in TCA cycle for ATP production has been reported in LPS stimulated macrophages. It has been suggested that this facilitates a variety of biosynthetic processes that depend on these intermediates (O'Neill, 2011; Tannahill et al., 2013; Lampropoulou et al., 2016; Mills et al., 2016). For example, citrate itaconic accumulates due to breaks in the TCA cycle between the isocitrate and alpha-ketoglutarate conversion step (catalyzed by isocitrate dehydrogenase) (Infantino et al., 2013; Jha et al., 2015). Citrate can also be replenished by the anaplerotic reactions of the TCA cycle (conversion of aspartate into oxaloacetate by aspartate aminotransferase). Export of citrate by the mitochondrial citrate carrier is accompanied by uptake of malate from the cytoplasm (converted to citrate by enzymes of the TCA cycle). In support of this possibility, a marked increase in transcripts for the citrate carrier was noted in LPS stimulated BMDCs. Furthermore, studies of macrophages demonstrate that LPS stimulation increased expression of the citrate carrier which increased cytosolic citrate levels which through the action of citrate lyase produces acetyl CoA and oxaloacetate. Oxoalocetate is converted to malate via malate dehydrogenase then pyruvate to generate NADPH required for ROS and NO production. Using gene silencing to ablate the citrate carrier gene in this system was therefore found to reduce NO and ROS production (Infantino et al., 2011). The concurrently generated aceyl-CoA can augment fatty acid synthesis (Everts et al., 2012; O'Neill and Pearce, 2016). Consequently, these changes to energy metabolism with accompanying biosynthetic reprogramming are likely to have a widespread influence on proximal and distal immune cell function including cytokine production and membrane remodeling. We suggest that these changes, similar to those seen following LPS stimulation (in the absence of infection) are host evolved mechanisms that occur during *T. gondii* infection are likely to provide host benefits in terms of shaping the immune response but can potentially aid parasite growth.

As the TCA cycle is linked to the ETC in mitochondria we examined the effect of *T. gondii* infection on transcripts for components of the ETC. We found that a number of these transcripts encoding complex I, II and III were down regulated in *T. gondii* infected BMDCs and LPS stimulated BMDCs. However, transcripts encoding complex IV and V subunits were increased in BMDCs infected *T. gondii* or stimulated with LPS. It has been observed in the literature that specific components of the ETC have additional roles other than generating energy including heme A biosynthesis, ion transport, calcium homeostasis, vesicle formation and lipid signaling (Antonicka et al., 2003; Sharma et al., 2019). Interestingly, there was an increase in gamma-glutamyl cysteine and cystine but not glutathione. These results suggest a response to oxidative stress (e.g., increased cysteine and glutathione synthesis to maintain intracellular glutathione levels. Additionally, to further determine the functional implications of these transcriptional changes we exploited the membrane potential-sensitive dye, TMRM. Our results indicate that mitochondria membrane potential is similarly down regulated in BMDCs infected with *T. gondii* or stimulated with LPS (Zhu et al., 2015; Van den Bossche et al., 2016). This is likely due to a reduced TCA cycle.

Changes to arginine metabolism in *T. gondii* infected BMDCs were also clearly evident in these studies described. Notably *T. gondii* infection favored the expression of arginase and production of L-ornithine, L-proline, L-1-pyrroline-3-hydroxy-5-carboxylate, and L-glutamate. Concomitant increases in the levels of transcript for *srm* which encodes spermidine synthesis was elevated in these infected cells suggesting further metabolism of ornthine to polyamines. In contrast LPS activation of BMDCs favored the expression of iNOS and the production of nitric oxide and citrulline. Opposing hypotheses can be formed as to whether these changes benefit the host or the parasite. Viewing the alteration in arginine metabolism as a host evolved strategy, arginine depletion has the potential to restrict *T. gondii* growth as it is an arginine auxotroph (Fox et al., 2004). Alternatively, directing arginine metabolism toward ornithine, proline and polyamines can also be viewed as a parasite evolved strategy subverting arginine metabolism toward arginase degradation reducing the ability of iNOS to produce nitric oxide which has been shown to have limit parasite multiplication. Additionally, diverting arginine toward ornithine and polyamines and ultimately to glutamate may provide *T. gondii* this resource for downstream use in the TCA cycle. In support of this studies have demonstrated that ROP16 (released from the rhoptry organelles during invasion) induces Arg expression in a STAT6 dependent manner (El Kasmi et al., 2008; Butcher et al., 2011; Marshall et al., 2011). In reality, as parasites and hosts evolve together both these hypotheses can coexist. Importantly, our previously published work demonstrates that *T. gondii* growth can be curtailed *in vivo* both through arginase dependent and iNOS dependent mechanisms (Woods et al., 2013).

Overall the studies described herein, demonstrate that the metabolism of BMDCs are profoundly affected by *T. gondii* infection in a manner similar to the Warburg effect. The multi-omics approach used here provides a wealth of metabolic and transcriptomic indicators of other pathways that are affected including arginine metabolism and provide insight in to the potential interactions of the host and parasite biochemistry. Understanding the interplay of these host and parasite interactions could provide insight into novel antimicrobial therapies including host directed interventions to limit parasite multiplication and survival.

## Data Availability

The RNASeq datasets generated for this study can be found in Figshare, https://doi.org/10.6084/m9.figshare.9751904.v1.

## Ethics Statement

All animal care and experimental procedures were conducted in accordance with relevant guidelines and regulations with the approval of the University of Strathclyde Animal Welfare and Ethical Review Body (AWERB), under UK Home Office regulations (Animals (Scientific Procedures) Act 1986, UK). Animals are housed according to or above the standard of the Home Office Code of Practice for the housing and care of animals bred, supplied or used for scientific purposes. The Appendix D Schedule 1 procedure dislocation of the neck was used to obtain tissue. This is followed by a confirmation method.

## Author Contributions

KH, OM, and CR conceived the study and participated in its design. KH with help from SW, co-cultured BMDCs with *Toxoplasma gondii* and coordinated all experiments described herein. SW performed the CBA assay. GW processed all the raw metabolomics and transcriptomics data-sets whilst KH analyzed and interpreted them. Technical help from SC was necessary to image the mitochondrial membrane potential of the *T. gondii* co-cultured BMDCs. KH performed statistical analysis and interpreted the results alongside CR. KH and CR wrote the manuscript. All authors read and approved the final manuscript.

### Conflict of Interest Statement

The authors declare that the research was conducted in the absence of any commercial or financial relationships that could be construed as a potential conflict of interest.
