# Supplementary material for: Multi-Omics Studies Demonstrate Toxoplasma gondii-Induced Metabolic Reprogramming of Murine Dendritic Cells
Source: Front Cell Infect Microbiol. 2019 Sep 11;9:309. doi: 10.3389/fcimb.2019.00309 (PMC6749083; doi:10.3389/fcimb.2019.00309)
Supplement: Supplementary file 1 [file Data_Sheet_1.docx]

Supplementary Material

## Table 1. Pairwise analysis of transcripts between *T. gondii* infected and naïve BMDC cultures.

| Transcript abbreviation  *T. gondii* compared to UN | M4.VIP[1+3+0]**^1^** | 2.57059 * M4.VIP[1]cvSE**^2^** |
| --- | --- | --- |
| Pde1c | 29.7705 | 23.2439 |
| Gm7665 | 28.7759 | 19.2809 |
| Gm7204 | 26.5943 | 23.1214 |
| Il1rapl1 | 26.3048 | 21.7306 **^3^** |
| Mir6236 | 24.518 | 20.1035 |
| Bbx | 18.925 | 12.5943 |
| Lyz2 | 18.752 | 19.3814 |
| Mir6240 | 17.297 | 16.1975 |
| Gm23374 | 16.1579 | 3.90884 |
| Tpt1-ps3 | 14.1425 | 8.14689 |
| Cntnap2 | 13.014 | 11.6975 |
| Ctcf | 10.6099 | 7.63508 |
| Gm8984 | 9.92352 | 5.78935 |
| Uqcrh-ps2 | 9.15427 | 5.72923 |
| Gm37361 | 8.68752 | 4.30196 |
| Rps28 | 8.63668 | 9.30479 |
| Gm26981 | 8.60619 | 2.50468 |
| Rpl27a-ps2 | 8.55443 | 6.42522 |
| Rnf24 | 8.4904 | 3.15227 |
| Hormad2 | 8.43401 | 5.27322 |
| Gm14303 | 8.25401 | 10.1217 |
| Fth1 | 8.20817 | 11.0134 |
| Gm5870 | 8.18754 | 4.45409 |
| Tcea1 | 8.06605 | 6.9676 |
| Pcsk5 | 7.94417 | 4.11131 |
| Cd74 | 7.84299 | 3.80337 |
| Gm43841 | 7.53214 | 11.6668 |
| Grm8 | 7.33362 | 2.25636 |
| Oaz1-ps | 7.16246 | 2.25568 |
| Gm11560 | 7.14158 | 7.66582 |
| Negr1 | 7.11428 | 4.10265 |
| Gm7846 | 7.04815 | 6.73632 |
| Cdk8 | 7.03808 | 6.73842 |
| Gm10180 | 7.029 | 6.3423 |
| Gm16418 | 6.99669 | 8.061 |
| Gm8909 | 6.97261 | 8.41092 |
| Gm12864 | 6.95786 | 6.18829 |
| Gm6394 | 6.92172 | 6.94704 |
| Gm13450 | 6.8032 | 6.15994 |
| G6pd2 | 6.65068 | 5.31818 |
| Macrod2 | 6.43796 | 5.34899 |
| Gm12111 | 6.4306 | 6.47916 |
| Bach2 | 6.332 | 5.25935 |
| 9330179D12Rik | 6.28371 | 8.66867 |
| Gm6682 | 6.24743 | 9.16653 |
| Mvp | 6.20346 | 6.42487 |
| Rpl3-ps1 | 6.19751 | 4.98168 |
| Tmcc1 | 6.10234 | 5.60922 |
| Cntnap5b | 6.0119 | 5.54144 |
| Gm15487 | 6.00558 | 9.20558 |
| S100a16 | 5.98421 | 3.53761 |
| Hs6st3 | 5.90294 | 7.5743 |
| Gm11822 | 5.8708 | 2.40601 |
| Cacna2d4 | 5.82441 | 5.30595 |
| Mmp12 | 5.82363 | 4.12476 |
| Gm12497 | 5.79329 | 4.86386 |
| Antxr1 | 5.76625 | 9.19581 |
| Gm12892 | 5.71355 | 7.31651 |
| Gm26745 | 5.66327 | 5.56723 |
| Gm24951 | 5.65057 | 5.84737 |
| Ldha | 5.64679 | 2.93692 |
| Gm7984 | 5.6226 | 5.32374 |
| Gm5809 | 5.60578 | 7.64309 |
| Rpl10l | 5.50105 | 1.72889 |
| Gm6987 | 5.46291 | 5.20976 |
| Vmn2r-ps54 | 5.46176 | 2.8816 |
| Gm15925 | 5.46 | 1.94196 |
| Zfp839 | 5.44477 | 6.92873 |
| Gm9575 | 5.33539 | 1.8852 |
| Anks1b | 5.24789 | 7.44367 |
| Gm2522 | 5.21617 | 3.13893 |
| Gm38187 | 5.19813 | 3.00196 |
| Gm16372 | 5.1112 | 3.57602 |
| H3f3a-ps2 | 5.0413 | 5.27278 |
| Gm6180 | 5.01923 | 5.42147 |
| Eif4a-ps4 | 5.01865 | 7.56183 |
| Gm43972 | 5.00667 | 5.65328 |
| Gm14130 | 4.98418 | 5.00535 |
| Actg1 | 4.95565 | 2.39176 |
| Gm7332 | 4.90504 | 3.54579 |
| Gm15173 | 4.89639 | 6.01423 |
| Txnrd1 | 4.77871 | 2.95159 |
| Zfp36l1-ps | 4.76386 | 1.20818 |
| Gm44090 | 4.76187 | 4.77858 |
| Ftl1 | 4.76101 | 3.59679 |
| Mir703 | 4.72025 | 4.25601 |
| Gm2824 | 4.71066 | 3.24511 |
| Camk1d | 4.7083 | 5.11768 |
| H2-Eb1 | 4.68394 | 1.80401 |
| Gm15482 | 4.65823 | 5.26864 |
| Gm12906 | 4.59467 | 2.99649 |
| Gm19087 | 4.55338 | 6.23466 |
| RP23-96L20.1 | 4.54006 | 6.54513 |
| Gm13453 | 4.53788 | 5.14045 |
| H2-Aa | 4.53547 | 3.1295 |
| Fam19a3 | 4.53028 | 3.95316 |
| Il31ra | 4.51987 | 3.82526 |
| Gm11970 | 4.51889 | 5.67451 |
| RP23-166L22.1 | 4.51419 | 5.20508 |
| Itfg1 | 4.4906 | 6.54473 |
| Gm13736 | 4.45762 | 5.57017 |
| Gm14877 | 4.43218 | 3.0881 |
| Lrrc20 | 4.41766 | 5.30971 |
| Rn18s-rs5 | 4.40228 | 2.36714 |
| Plxdc1 | 4.39943 | 3.43812 |
| Oca2 | 4.38513 | 6.58284 |
| Hp | 4.37617 | 1.42688 |
| Gm9025 | 4.3421 | 5.27872 |
| Cntnap2 | 4.34151 | 3.95479 |
| Ctsd | 4.3234 | 3.49933 |
| Trim30e-ps1 | 4.30239 | 2.1315 |
| Gm3145 | 4.29543 | 5.10707 |
| Gm10698 | 4.26661 | 4.00935 |
| Lrp1b | 4.25801 | 7.4716 |
| Sh2d2a | 4.22432 | 4.54484 |
| Gm8048 | 4.21024 | 2.5951 |
| Tpt1-ps6 | 4.18195 | 3.26607 |
| Scp2-ps2 | 4.1585 | 6.23664 |
| Mup-ps16 | 4.14291 | 4.93893 |
| Tmem232 | 4.13982 | 4.39107 |
| Angel1 | 4.13494 | 6.91054 |
| Slc39a1-ps | 4.12768 | 5.41029 |
| Gm7634 | 4.12577 | 4.54922 |
| Gm8304 | 4.11228 | 5.30512 |
| Prdx1 | 4.10223 | 1.80965 |
| Lgals3 | 4.09547 | 1.88656 |
| Gm4852 | 4.05341 | 4.23926 |
| Rnft2 | 4.05179 | 5.10186 |
| Ccl22 | 4.04173 | 1.66659 |
| Gm44978 | 4.01591 | 2.77378 |
| Gm10221 | 4.0134 | 3.47887 |
| Gm8529 | 4.00488 | 5.44795 |
| Spp1 | 3.98866 | 1.32201 |
| Gm29610 | 3.98571 | 3.92216 |
| Coro1a | 3.97979 | 0.700581 |
| Rpl36a-ps1 | 3.97777 | 5.96198 |
| Ralgps2 | 3.96167 | 2.71459 |
| Gm44737 | 3.95973 | 3.44625 |
| Gpx1 | 3.95636 | 2.65059 |
| Clec4n | 3.95573 | 5.77471 |
| Myo9a | 3.95026 | 3.59838 |
| Gm15529 | 3.92407 | 2.17757 |
| Gm35106 | 3.91488 | 3.14183 |
| Usp32 | 3.90663 | 5.7439 |
| Gm7676 | 3.88365 | 2.40948 |
| 2410017I17Rik | 3.86103 | 4.96845 |
| Lpl | 3.85776 | 3.01992 |
| Eef1a1 | 3.84943 | 2.5738 |
| Chd2 | 3.84328 | 2.82575 |
| Tmsb4x | 3.84058 | 4.35322 |
| Gm6863 | 3.82726 | 3.00587 |
| Sepw1 | 3.78945 | 3.9936 |
| Gm16755 | 3.77349 | 4.53077 |
| Rpl9-ps8 | 3.77246 | 2.2026 |
| Mt1 | 3.76974 | 1.35457 |
| Rit2 | 3.76368 | 4.87941 |
| Crocc | 3.75711 | 2.80243 |
| Ctss | 3.74291 | 2.51818 |
| Gm42989 | 3.73269 | 6.14086 |
| Gm11258 | 3.73191 | 5.32677 |
| Gm5560 | 3.70122 | 6.75786 |
| 4833427G06Rik | 3.67907 | 2.52195 |
| Nckap5 | 3.67728 | 3.06549 |
| Gm16111 | 3.6763 | 4.8957 |
| Gm16407 | 3.67372 | 5.22696 |
| Trim28 | 3.6581 | 3.35552 |
| Frem1 | 3.62962 | 2.33604 |
| Gm11951 | 3.61937 | 2.66448 |
| Eif1ax | 3.60366 | 3.68303 |
| H2-Ab1 | 3.59643 | 1.39966 |
| Gm5548 | 3.58819 | 6.18967 |
| Gm7293 | 3.58123 | 1.13832 |
| Rps14 | 3.57689 | 3.14828 |
| Tcp1 | 3.57533 | 3.35064 |
| Gm7964 | 3.56283 | 4.73159 |
| H2-Ea-ps | 3.55803 | 3.32547 |
| Gm2199 | 3.53226 | 4.48624 |
| Gm609 | 3.51457 | 3.6672 |
| Gm37945 | 3.51039 | 2.47147 |
| Slc25a5-ps | 3.48172 | 5.1535 |
| mt-Nd6 | 3.47737 | 4.26539 |
| Gm6913 | 3.47064 | 5.28359 |
| Gm26825 | 3.45706 | 2.61038 |
| Gm26659 | 3.45699 | 3.79212 |
| Mmp9 | 3.44703 | 0.754628 |
| Gm42418 | 3.44556 | 2.35034 |
| Lrba | 3.44167 | 3.95871 |
| Gm9840 | 3.42667 | 1.07144 |
| Gm38305 | 3.42039 | 3.73919 |
| mt-Cytb | 3.40916 | 3.93789 |
| Batf2 | 3.40843 | 4.4449 |
| Cotl1 | 3.39753 | 2.39081 |
| Slc28a1 | 3.36566 | 4.70904 |
| Gm42522 | 3.36538 | 2.54249 |
| 4930552N02Rik | 3.36231 | 6.17561 |
| Rps5 | 3.36002 | 2.48686 |
| Ccdc7b | 3.35309 | 1.6141 |
| Gm44209 | 3.35282 | 2.85952 |
| Gm20589 | 3.31998 | 3.28937 |
| Gm9824 | 3.31583 | 6.60793 |
| Gm13132 | 3.29796 | 2.55142 |
| Gm28343 | 3.29792 | 6.35634 |
| Gm18848 | 3.28422 | 1.1003 |
| Gm12428 | 3.2793 | 3.07351 |
| Ifitm1 | 3.27594 | 2.09789 |
| Gnaq | 3.27537 | 3.93779 |
| Gm10814 | 3.26834 | 3.41308 |
| Gm9159 | 3.26605 | 2.22355 |
| Syngr2 | 3.26125 | 0.883411 |
| Prdx5 | 3.25833 | 2.98722 |
| Gm4374 | 3.25167 | 3.47938 |
| Gm7335 | 3.24404 | 3.02952 |
| Tcf12 | 3.23635 | 3.64253 |
| Magee2 | 3.22996 | 5.21722 |
| Gm5244 | 3.2264 | 4.41611 |
| Eef2 | 3.22635 | 2.17653 |
| RP23-457I3.2 | 3.22534 | 4.97695 |
| Acyp2 | 3.2238 | 3.66693 |
| Fcrla | 3.21823 | 2.03935 |
| Usp37 | 3.20715 | 0.977969 |
| Gm20412 | 3.20043 | 0.707146 |
| Gm17638 | 3.20017 | 0.763761 |
| Rplp1 | 3.19469 | 3.27905 |
| Zfyve28 | 3.17322 | 1.64631 |
| RP23-183A4.1 | 3.17225 | 6.71829 |
| Gm6265 | 3.16873 | 3.17535 |
| Gm5940 | 3.16044 | 4.30964 |
| Rps9 | 3.15775 | 2.43598 |
| Gm15703 | 3.1503 | 4.00203 |
| Sepp1 | 3.14895 | 1.23889 |
| Gm36551 | 3.14232 | 4.14277 |
| Gm26530 | 3.13706 | 0.788046 |
| Rps18 | 3.13281 | 2.37131 |
| Tubb5 | 3.11681 | 1.28341 |
| Rps29 | 3.11244 | 3.53373 |
| Ryr3 | 3.10459 | 4.34159 |
| Mt2 | 3.10096 | 0.566107 |
| Gm15645 | 3.09509 | 3.75885 |
| Gm6788 | 3.09125 | 4.5796 |
| Sirpb1b | 3.07948 | 3.28369 |
| Gm38015 | 3.05446 | 2.80662 |
| Tpt1 | 3.03868 | 2.78492 |
| RP23-297C11.2 | 3.02961 | 2.71333 |
| Gm8814 | 3.02691 | 2.83466 |
| Prex2 | 3.01209 | 3.47267 |
| Sh3gl3 | 3.00789 | 1.844 |
| Ebf1 | 3.00191 | 4.11093 |
| Hhat | 2.99936 | 2.33029 |
| Gm19587 | 2.99349 | 4.30423 |
| Gm5615 | 2.99103 | 2.12047 |
| Rack1 | 2.97926 | 2.07832 |
| Pou2f3 | 2.97254 | 4.36922 |
| Gm43495 | 2.9704 | 2.74603 |
| Csf1r | 2.96061 | 0.765463 |
| Gm16589 | 2.94774 | 2.18388 |
| Saa3 | 2.93209 | 3.00792 |
| Gm20511 | 2.92113 | 4.14412 |
| Capg | 2.91775 | 2.07158 |
| Ccni | 2.90955 | 1.08531 |
| Sh3bgrl | 2.90034 | 0.843186 |
| Ctsb | 2.89716 | 0.936777 |
| 6030443J06Rik | 2.89444 | 3.0006 |
| 4930506C21Rik | 2.88868 | 1.22332 |
| Gpnmb | 2.88479 | 0.790067 |
| Gm43083 | 2.88189 | 1.37842 |
| Gm12017 | 2.8091 | 1.51908 |
| Gm12125 | 2.78314 | 1.6283 |
| Rps10 | 2.777 | 2.29398 |
| Gm13392 | 2.76123 | 5.04857 |
| Spock3 | 2.74958 | 1.47045 |
| Gm5578 | 2.74881 | 4.57025 |
| Gm36938 | 2.74568 | 2.66601 |
| Aldoart1 | 2.745 | 2.50499 |
| mt-Nd5 | 2.73599 | 3.68155 |
| Ptma | 2.7327 | 2.07598 |
| B830042I05Rik | 2.73223 | 2.71576 |
| Gm5997 | 2.72379 | 5.43495 |
| Gm5315 | 2.71067 | 3.05149 |
| Tyrobp | 2.71048 | 1.89888 |
| Cd36 | 2.70668 | 0.915411 |
| Gm22553 | 2.70144 | 2.97563 |
| Gm18957 | 2.69651 | 3.66086 |
| RP23-74O12.6 | 2.67539 | 2.79426 |
| Atp5e | 2.66807 | 0.786915 |
| Txlnb | 2.66169 | 2.74303 |
| Rps19 | 2.66123 | 2.27991 |
| Lgals1 | 2.65136 | 0.598193 |
| Gm5124 | 2.6419 | 2.18558 |
| Gm5871 | 2.63452 | 1.03473 |
| Agtpbp1 | 2.62464 | 4.02059 |
| Txnrd1 | 2.62018 | 2.69631 |
| Gm14204 | 2.6183 | 1.9697 |
| Ccl6 | 2.61685 | 0.723318 |
| Zyx | 2.61578 | 0.692749 |
| Tgm2 | 2.59514 | 0.463243 |
| Gm26873 | 2.57344 | 3.53754 |
| Gm26561 | 2.56825 | 2.12063 |
| 4930509G22Rik | 2.55814 | 4.97498 |
| Zbtb20 | 2.54878 | 2.96127 |
| Cks1brt | 2.54724 | 3.00336 |
| Gm42535 | 2.54165 | 2.90771 |
| Gm19587 | 2.54141 | 1.45803 |
| Lhx9 | 2.53884 | 1.50163 |
| Erbb4 | 2.53552 | 3.30369 |
| Asap2 | 2.53238 | 4.41624 |
| Gm17087 | 2.52596 | 1.43899 |
| Itm2b | 2.51577 | 1.51591 |
| Gm8801 | 2.51532 | 0.661912 |
| Laptm5 | 2.51344 | 1.14121 |
| Ighv1-64 | 2.5043 | 2.94832 |
| Anxa1 | 2.50381 | 1.91332 |
| Pla2g7 | 2.50066 | 1.18629 |
| Trim2 | 2.49666 | 3.22072 |
| Rhbdd3 | 2.48524 | 1.54749 |
| Gm19963 | 2.47428 | 2.84547 |
| Fn1 | 2.46758 | 3.31568 |
| Slk | 2.44651 | 2.48834 |
| Gm4076 | 2.44593 | 1.36253 |
| Gm28196 | 2.44473 | 2.52581 |
| H2-Bl | 2.42743 | 1.74633 |
| Gm1840 | 2.41336 | 2.91487 |
| Arg1 | 2.40537 | 1.0499 |
| Gm17909 | 2.40506 | 1.03824 |
| Loxl3 | 2.40437 | 0.642673 |
| Txnrd1 | 2.40084 | 2.38725 |
| RP24-426B21.1 | 2.39807 | 3.2292 |
| Ccl9 | 2.38185 | 0.926765 |
| Pou3f4 | 2.37735 | 3.5034 |
| Txnrd1 | 2.37461 | 1.33565 |
| Gm26852 | 2.37444 | 1.55096 |
| Cybb | 2.37179 | 0.517222 |
| Cox4i1 | 2.36546 | 2.16626 |
| Tpi1 | 2.34359 | 2.4009 |
| Gm16575 | 2.32971 | 1.36631 |
| Gm7291 | 2.32527 | 3.10158 |
| Rpl18a | 2.32418 | 2.00522 |
| Rpl26 | 2.31261 | 1.29894 |
| Anxa3 | 2.30792 | 0.800649 |
| Gm26586 | 2.3001 | 1.59325 |
| Tmsb10 | 2.29201 | 3.56717 |
| Hspe1-ps6 | 2.29022 | 2.89707 |
| Rps17 | 2.28837 | 2.26792 |
| Mei4 | 2.28722 | 2.76123 |
| Tmem123 | 2.28181 | 2.27334 |
| Gm9825 | 2.28176 | 3.49502 |
| Gpc5 | 2.26249 | 3.59243 |
| Slc8a3 | 2.24725 | 3.09118 |
| Tmprss11b | 2.2471 | 2.50304 |
| Gm18367 | 2.23843 | 1.5239 |
| Cep72 | 2.23698 | 2.17489 |
| Cyba | 2.23367 | 1.83855 |
| Ptprb | 2.23364 | 1.15693 |
| Gm37103 | 2.23274 | 1.83368 |
| Clec7a | 2.23079 | 3.46261 |
| Gm13502 | 2.22676 | 0.967478 |
| Ly6e | 2.22657 | 1.08732 |
| Gm43942 | 2.21284 | 1.14906 |
| Fpr2 | 2.21106 | 1.05546 |
| Myl6 | 2.20932 | 1.7448 |
| Hmox1 | 2.20001 | 3.12712 |
| Gm7935 | 2.1964 | 1.94073 |
| Gm15932 | 2.19472 | 1.67094 |
| Sema3a | 2.19388 | 2.34419 |
| Txnip | 2.18916 | 1.29229 |
| Rps12 | 2.18554 | 2.46301 |
| Lars2 | 2.18554 | 1.85184 |
| Gm13709 | 2.1756 | 0.431107 |
| Gsn | 2.16595 | 2.49548 |
| Mmp8 | 2.16047 | 1.48866 |
| Gm10390 | 2.15856 | 1.66479 |
| Gm15603 | 2.15682 | 1.8342 |
| Gm6166 | 2.15462 | 0.940891 |
| Glipr1 | 2.15329 | 0.737688 |
| Gm38033 | 2.15169 | 3.44981 |
| Unc93b1 | 2.14626 | 0.961251 |
| Emp3 | 2.14024 | 1.57349 |
| Sfi1 | 2.13677 | 1.76918 |
| Fam227b | 2.13535 | 2.21682 |
| Lipa | 2.12784 | 1.57197 |
| Gm8425 | 2.12748 | 0.959372 |
| Gm12352 | 2.11994 | 3.19424 |
| Gm14849 | 2.10247 | 3.15522 |
| Pfn1 | 2.10188 | 1.44442 |
| Gm27747 | 2.10147 | 2.89165 |
| Calr | 2.09312 | 1.1859 |
| Rps21 | 2.09295 | 1.20812 |
| Gm42801 | 2.08739 | 2.27046 |
| Cfb | 2.08659 | 0.531291 |
| P4hb | 2.08584 | 3.19934 |
| H2afj | 2.08115 | 0.592108 |
| Gm26819 | 2.07907 | 1.46975 |
| Slc3a2 | 2.07798 | 2.13197 |
| Kifc5c-ps | 2.07531 | 2.44581 |
| Pcbp2 | 2.07512 | 1.28986 |
| Cfp | 2.07394 | 1.62126 |
| Fau | 2.07386 | 2.24204 |
| Rps19-ps10 | 2.07298 | 3.52639 |
| Ccdc7a | 2.0727 | 1.88345 |
| Ctsh | 2.06902 | 1.55617 |
| Wfdc17 | 2.06753 | 1.25129 |
| Pkm | 2.06153 | 3.26901 |
| Actr3 | 2.05916 | 0.71206 |
| Fxyd5 | 2.05645 | 3.36338 |
| Tacr3 | 2.04815 | 1.37777 |
| Crb1 | 2.0417 | 2.03847 |
| Cd9 | 2.03685 | 0.448327 |
| Gm13361 | 2.03412 | 2.2152 |
| Gm14912 | 2.03344 | 2.51531 |
| Ctsz | 2.02721 | 0.432684 |
| Gm13365 | 2.02545 | 1.24368 |
| Srgn | 2.02506 | 2.20766 |
| Gm7816 | 2.02118 | 1.36828 |
| Rpl8 | 2.01887 | 2.3688 |
| Il1rn | 2.01829 | 2.65501 |
| Txnrd1 | 2.01797 | 2.95835 |
| Atp6v1b2 | 2.01399 | 1.36923 |
| Gm17275 | 2.01061 | 2.45762 |
| Gm13082 | 2.0062 | 4.33137 |
| Vapb | 2.00349 | 0.531742 |
| Rpl32 | 2.00223 **^4^** | 1.5237 |

## Table 2. Pairwise analysis of transcripts between LPS stimulated and naïve BMDC cultures.

| Transcript abbreviation | M5.VIP[1+2+0]**^1^** | 2.57059 * M5.VIP[1]cvSE**^2^** |
| --- | --- | --- |
| Mctp2 | 22.0702 | 4.80413 |
| Gm7665 | 21.7438 | 11.6129 |
| Gm7204 | 20.3053 | 13.1787 |
| Il1rapl1 | 16.0886 | 6.48698 **^3^** |
| Mir6236 | 15.3491 | 7.46573 |
| Rps28 | 13.5543 | 7.66528 |
| Gm7897 | 13.0526 | 1.9577 |
| Lyz2 | 12.9911 | 1.85868 |
| Mir6240 | 12.8504 | 20.4726 |
| Pde1c | 12.7338 | 16.52 |
| Ptprb | 12.4172 | 2.37236 |
| Bbx | 12.3005 | 16.8467 |
| Il1b | 11.8522 | 1.49574 |
| Gm23374 | 11.2611 | 5.78903 |
| Gm8909 | 11.065 | 9.40863 |
| Txlnb | 10.9932 | 2.65231 |
| Saa3 | 10.0443 | 2.32692 |
| Gm15487 | 9.67846 | 4.90614 |
| Bach2 | 9.59113 | 2.67182 |
| Gm14303 | 9.52444 | 3.3843 |
| Slc8a3 | 9.46479 | 6.41463 |
| Ftl1 | 9.06359 | 3.66487 |
| Ccl3 | 8.77091 | 0.671771 |
| Fam19a3 | 8.4366 | 6.8673 |
| Fth1 | 8.16427 | 9.03162 |
| Il1a | 8.14683 | 1.00103 |
| Ccl5 | 7.89059 | 1.83975 |
| Negr1 | 7.85983 | 6.07063 |
| Gm26745 | 7.84256 | 3.80377 |
| Tpt1-ps3 | 7.74534 | 8.87472 |
| Lhx9 | 7.73474 | 2.62715 |
| Rsad2 | 7.66712 | 1.28333 |
| Gm26615 | 7.64091 | 3.44969 |
| Cacna2d4 | 7.55598 | 3.124 |
| Hormad2 | 7.48156 | 11.855 |
| Cxcl3 | 7.48058 | 0.939714 |
| RP23-166L22.1 | 7.30342 | 4.77406 |
| Ctcf | 7.21433 | 6.12382 |
| Marcksl1 | 7.14277 | 0.68757 |
| Cntnap2 | 7.01352 | 4.95127 |
| Ccl22 | 6.96612 | 0.68272 |
| Ctsd | 6.80572 | 4.25124 |
| Gm26825 | 6.75372 | 2.37898 |
| Gm19963 | 6.73385 | 1.93428 |
| Timp4 | 6.64684 | 3.29144 |
| Il1rn | 6.50856 | 1.91968 |
| Gm16505 | 6.50137 | 2.50128 |
| Gm10425 | 6.40629 | 1.34229 |
| Eif1ax | 6.39948 | 5.59206 |
| Gm8984 | 6.37843 | 5.15289 |
| Gm7846 | 6.37261 | 3.9862 |
| Gm43841 | 6.34765 | 9.63577 |
| Batf2 | 6.17821 | 1.83577 |
| Gm16418 | 6.17146 | 5.46803 |
| Gm32444 | 6.07836 | 4.63169 |
| Gm11560 | 6.01328 | 8.28045 |
| Gm18957 | 5.96585 | 4.59768 |
| Vmn2r-ps54 | 5.88722 | 2.52573 |
| Gm12111 | 5.81265 | 5.06548 |
| Lgals3 | 5.8012 | 3.68418 |
| Rpl3-ps1 | 5.69433 | 2.58102 |
| Gm24951 | 5.61477 | 3.12648 |
| Gm17638 | 5.58299 | 3.37085 |
| Zfp36l1-ps | 5.57901 | 5.50155 |
| Usp32 | 5.56623 | 2.41646 |
| Pou2f3 | 5.43142 | 2.98944 |
| Gm8529 | 5.42166 | 4.24297 |
| Sh3gl3 | 5.38371 | 5.26674 |
| Gm7676 | 5.36873 | 4.37514 |
| Uqcrh-ps2 | 5.3558 | 4.41975 |
| Acod1 | 5.34843 | 0.497866 |
| Cd74 | 5.30519 | 1.44851 |
| Rit2 | 5.26721 | 4.30553 |
| Mir703 | 5.2332 | 2.28982 |
| Cxcl2 | 5.17602 | 0.587109 |
| Clec4n | 5.16185 | 2.24777 |
| Cdk8 | 5.15566 | 2.93539 |
| 4930552N02Rik | 5.09 | 3.41931 |
| Serpina3n | 5.07299 | 0.870231 |
| Gm7332 | 5.07283 | 2.66959 |
| Ccl4 | 5.07052 | 0.642739 |
| Isg15 | 5.05694 | 1.21715 |
| Il12b | 5.02507 | 0.249562 |
| Gm35106 | 5.01144 | 4.96362 |
| Anks1b | 5.00236 | 3.40691 |
| Eif4a-ps4 | 4.99003 | 4.98352 |
| 9330179D12Rik | 4.98448 | 6.674 |
| Hp | 4.97575 | 1.11623 |
| Ctss | 4.93679 | 2.98342 |
| Rnf24 | 4.91556 | 2.71245 |
| Gm8814 | 4.8721 | 0.921598 |
| Rn18s-rs5 | 4.86515 | 8.85417 |
| Gpx1 | 4.81536 | 1.54816 |
| Cmpk2 | 4.80964 | 0.754949 |
| Gm5870 | 4.73405 | 3.71078 |
| Gm45211 | 4.72901 | 1.23173 |
| AA467197 | 4.71926 | 1.25108 |
| H3f3a-ps2 | 4.68161 | 4.98012 |
| Gm10180 | 4.61995 | 3.5864 |
| Gm15925 | 4.616 | 1.69804 |
| Lcn2 | 4.61336 | 0.224425 |
| Fscn1 | 4.5963 | 1.02945 |
| Gm7634 | 4.55951 | 3.62055 |
| Eef1a1 | 4.52435 | 1.14882 |
| Macrod2 | 4.50138 | 5.12147 |
| Clec4e | 4.48615 | 1.14011 |
| Cxcl10 | 4.46435 | 0.457183 |
| Tcea1 | 4.45069 | 6.15291 |
| Aldoart1 | 4.42872 | 5.00991 |
| Mmp12 | 4.41911 | 4.31692 |
| Sepw1 | 4.41133 | 7.88075 |
| Gm609 | 4.3991 | 3.16189 |
| Gm13392 | 4.38426 | 9.18607 |
| Gm6863 | 4.36515 | 4.25726 |
| Ptgs2 | 4.3636 | 0.235512 |
| Gm12158 | 4.35452 | 4.75412 |
| Usp37 | 4.35308 | 3.34348 |
| Actg1 | 4.31708 | 0.796986 |
| H2-Q3 | 4.3103 | 2.0023 |
| Gm16589 | 4.30603 | 3.37432 |
| 4833427G06Rik | 4.26012 | 1.8037 |
| Prdx5 | 4.25794 | 2.96928 |
| Gm6394 | 4.2084 | 4.36168 |
| Gm26981 | 4.18815 | 1.96592 |
| Gm9824 | 4.17474 | 3.74528 |
| Cep72 | 4.17273 | 0.628737 |
| Gm13736 | 4.17054 | 1.73419 |
| 6030443J06Rik | 4.12441 | 5.03333 |
| Il6 | 4.11913 | 0.797053 |
| Gm5548 | 4.09999 | 2.13572 |
| Mt2 | 4.0952 | 0.7988 |
| RP23-183A4.1 | 4.07256 | 3.39732 |
| Serpina3g | 4.03763 | 0.586658 |
| Mmp13 | 4.02591 | 0.815249 |
| Tyrobp | 4.01295 | 1.98474 |
| Gbp2 | 4.00843 | 0.462921 |
| Cd40 | 3.99453 | 0.777081 |
| H2-Bl | 3.98634 | 4.50697 |
| G6pd2 | 3.97624 | 3.13266 |
| AW112010 | 3.9681 | 0.575498 |
| RP23-74O12.6 | 3.95426 | 2.00043 |
| Il31ra | 3.93047 | 4.67513 |
| Gbp2b | 3.92764 | 0.57648 |
| Gm6913 | 3.91291 | 4.10817 |
| Gm6682 | 3.90886 | 4.98436 |
| Crocc | 3.9011 | 4.27762 |
| Cpa6 | 3.88717 | 1.83706 |
| Eef2 | 3.8773 | 0.727642 |
| Pcsk5 | 3.87437 | 4.41553 |
| Rplp1 | 3.87324 | 2.05142 |
| Gm12892 | 3.86935 | 5.17795 |
| Gm42989 | 3.86429 | 3.75413 |
| Tmem232 | 3.85915 | 2.88169 |
| Oasl1 | 3.85645 | 0.386414 |
| Camk1d | 3.84676 | 6.3562 |
| Gm29610 | 3.84557 | 0.896652 |
| Cotl1 | 3.83888 | 1.59583 |
| Gm4585 | 3.83561 | 2.85326 |
| Gm5578 | 3.82587 | 3.39583 |
| Cntnap5b | 3.79971 | 3.56663 |
| Gm5607 | 3.78758 | 1.1937 |
| Mvp | 3.77658 | 4.44171 |
| B830042I05Rik | 3.76287 | 1.84582 |
| Oaz1-ps | 3.75452 | 6.04302 |
| Tmcc1 | 3.72408 | 2.03723 |
| Spp1 | 3.70178 | 3.87178 |
| Nckap5 | 3.68878 | 2.39833 |
| Ccl9 | 3.67012 | 3.47673 |
| Gm9089 | 3.66666 | 1.7213 |
| Sema3a | 3.66173 | 5.28868 |
| Gm9025 | 3.66104 | 4.67956 |
| Gm15173 | 3.6468 | 3.4604 |
| Gm28539 | 3.62501 | 2.97957 |
| Gm37103 | 3.61535 | 1.7364 |
| Lrrc20 | 3.61457 | 5.47451 |
| Plek | 3.60782 | 0.847415 |
| Rps18 | 3.59692 | 1.62441 |
| Clic4 | 3.59041 | 0.445724 |
| Sepp1 | 3.58529 | 0.57123 |
| Gm28196 | 3.56717 | 4.07747 |
| Gm20470 | 3.55425 | 0.692599 |
| Gm7816 | 3.54085 | 2.52019 |
| S100a16 | 3.53959 | 3.0332 |
| Csf1r | 3.5183 | 0.256595 |
| Gm5560 | 3.50735 | 3.37848 |
| Gm11970 | 3.50514 | 2.87821 |
| Socs3 | 3.50237 | 0.189562 |
| 2410017I17Rik | 3.49657 | 2.94009 |
| Cxcl1 | 3.49553 | 0.29737 |
| Plxdc1 | 3.49071 | 5.66769 |
| Serpine1 | 3.48596 | 0.425144 |
| Vim | 3.48157 | 0.961071 |
| Dmd | 3.47511 | 4.58991 |
| Gm11258 | 3.46996 | 5.22512 |
| Gm44090 | 3.46531 | 2.75824 |
| Coro1a | 3.46343 | 0.36377 |
| Gm19587 | 3.45462 | 3.09517 |
| Pnp | 3.45329 | 1.33662 |
| Rps14 | 3.44457 | 2.18043 |
| Rpl32 | 3.44113 | 1.65154 |
| Gm5244 | 3.44083 | 2.42039 |
| Slk | 3.43613 | 2.6815 |
| Txn1 | 3.43104 | 1.25686 |
| Cd36 | 3.42787 | 2.7859 |
| Irf7 | 3.42754 | 0.778798 |
| Fxyd5 | 3.4196 | 1.35697 |
| Gm44737 | 3.41591 | 1.39366 |
| Pkm | 3.41064 | 1.41173 |
| Scp2-ps2 | 3.40902 | 2.76861 |
| Ccl6 | 3.3998 | 2.4682 |
| Rps5 | 3.37858 | 1.51743 |
| Crb1 | 3.37623 | 2.50465 |
| Lamp1 | 3.37295 | 0.452457 |
| Rps19-ps10 | 3.36333 | 1.81186 |
| Txnrd1 | 3.3553 | 1.48357 |
| Erbb4 | 3.3546 | 4.32679 |
| Slc15a3 | 3.35025 | 0.767382 |
| H3f3b | 3.33796 | 0.530627 |
| Ebf1 | 3.33697 | 2.16784 |
| Ms4a6d | 3.33401 | 0.671548 |
| H2-Aa | 3.31398 | 1.70986 |
| Gm12428 | 3.31296 | 0.867835 |
| Gm26852 | 3.30875 | 2.78979 |
| Zfp839 | 3.30065 | 3.30031 |
| Rpl8 | 3.29756 | 1.16527 |
| Slc2a6 | 3.28998 | 0.551542 |
| Tpt1 | 3.28235 | 1.87407 |
| Zfp956 | 3.27602 | 0.50509 |
| Zfyve28 | 3.274 | 3.70641 |
| Apoe | 3.2724 | 1.00506 |
| Gm5940 | 3.26968 | 4.79248 |
| Rps9 | 3.26634 | 1.36666 |
| Upp1 | 3.25719 | 0.971944 |
| Cebpb | 3.25572 | 0.600858 |
| Lpl | 3.23954 | 3.14418 |
| Gm8304 | 3.2378 | 4.08211 |
| Laptm5 | 3.23656 | 0.263358 |
| Gm7293 | 3.2296 | 4.58769 |
| Pnp | 3.22292 | 0.653491 |
| Gm13502 | 3.21379 | 2.57604 |
| Atp5e | 3.21215 | 2.36349 |
| Fry | 3.20557 | 1.29696 |
| Cd14 | 3.18475 | 1.35672 |
| Oca2 | 3.1808 | 4.47946 |
| Gm3145 | 3.1704 | 4.29608 |
| Gm2670 | 3.16996 | 1.96766 |
| Ifit2 | 3.1668 | 0.327351 |
| Gm13450 | 3.14405 | 2.58875 |
| Tnfaip2 | 3.13978 | 0.201345 |
| Acsl1 | 3.13667 | 0.167353 |
| Gm36551 | 3.10755 | 3.10526 |
| Fn1 | 3.10634 | 2.73731 |
| Gm6180 | 3.09951 | 5.37202 |
| Gbp3 | 3.09634 | 0.424083 |
| Gm6987 | 3.08896 | 5.50969 |
| Psme2 | 3.08493 | 0.902506 |
| Gm4852 | 3.08398 | 4.89768 |
| Rps17 | 3.08019 | 1.38832 |
| Fam227b | 3.07984 | 0.54735 |
| F10 | 3.07888 | 0.524093 |
| Prdx1 | 3.07878 | 1.53504 |
| Pfn1 | 3.06827 | 1.77966 |
| S100a9 | 3.0671 | 2.70094 |
| Lipa | 3.06607 | 1.51157 |
| BC048502 | 3.06524 | 1.02872 |
| Ehd1 | 3.06382 | 0.357829 |
| Gm20589 | 3.05286 | 3.30845 |
| Retnla | 3.04887 | 1.63601 |
| Asap2 | 3.04572 | 2.7473 |
| Gm8979 | 3.04171 | 2.98705 |
| Gm12497 | 3.0356 | 3.70043 |
| Ifi47,Olfr56 | 3.0346 | 0.86441 |
| Gm43083 | 3.03139 | 0.903122 |
| Gm12060 | 3.0304 | 3.70268 |
| Gm28343 | 3.02311 | 3.94269 |
| Mmp8 | 3.02011 | 0.98913 |
| Rack1 | 3.01022 | 1.31122 |
| Rps11 | 3.00586 | 1.05231 |
| Slc7a2 | 3.00532 | 0.396886 |
| Slfn2 | 3.00334 | 0.864154 |
| Rps26 | 3.00306 | 0.913244 |
| Gm5615 | 2.99031 | 1.42287 |
| Ctsb | 2.9896 | 3.13614 |
| Rps16 | 2.98846 | 1.24276 |
| Gm42418 | 2.9881 | 5.27854 |
| Tagln2 | 2.97706 | 1.51111 |
| Gm5997 | 2.97247 | 3.9031 |
| Antxr1 | 2.97173 | 3.25881 |
| Rpl18a | 2.96856 | 0.885574 |
| Tnfrsf1b | 2.96115 | 0.0882285 |
| Nos2 | 2.95144 | 6.20242 |
| Kcp | 2.93074 | 2.72255 |
| Lcp1 | 2.91477 | 0.967603 |
| Usp18 | 2.91456 | 0.506088 |
| Nfkbia | 2.90698 | 0.427773 |
| Tnf | 2.90177 | 0.413997 |
| Rps19 | 2.90033 | 1.27147 |
| Pla2g7 | 2.89866 | 0.767175 |
| Gm4374 | 2.89407 | 1.68355 |
| Wfdc21 | 2.8924 | 0.984978 |
| Sdc4 | 2.89191 | 0.446792 |
| Id2 | 2.88865 | 0.688839 |
| Lgals1 | 2.87891 | 2.46663 |
| Itm2b | 2.87481 | 1.01916 |
| Rps12 | 2.87446 | 1.25276 |
| Mapkapk2 | 2.86619 | 0.391048 |
| Fshr | 2.86205 | 1.24873 |
| Tpt1-ps6 | 2.8615 | 2.11084 |
| Hck | 2.85841 | 0.581284 |
| Rps15 | 2.85702 | 1.35275 |
| Grm8 | 2.84842 | 2.14474 |
| Gm10221 | 2.84661 | 1.1567 |
| Gm20511 | 2.84038 | 2.61819 |
| Cks1brt | 2.83433 | 2.02921 |
| H2-Eb1 | 2.83311 | 1.2399 |
| Gm15703 | 2.82352 | 1.27497 |
| Rps4x | 2.81904 | 0.665833 |
| Anxa2 | 2.81896 | 1.38346 |
| Rps21 | 2.81356 | 2.16656 |
| Cdkn1a | 2.80542 | 0.384012 |
| Tapbp,Zbtb22 | 2.80264 | 0.453199 |
| Gm7335 | 2.79979 | 1.2279 |
| Rnft2 | 2.79898 | 1.8003 |
| Gm44978 | 2.79193 | 2.99615 |
| Mmp14 | 2.78056 | 0.296787 |
| Ifi44 | 2.77857 | 0.739124 |
| 4930509G22Rik | 2.77678 | 4.83223 |
| Lgals9 | 2.77629 | 0.688719 |
| Slc39a1-ps | 2.76883 | 4.01684 |
| Srgn | 2.76279 | 1.31715 |
| Spock3 | 2.75844 | 1.40919 |
| Rpl9 | 2.73605 | 1.15298 |
| P4hb | 2.73523 | 1.15321 |
| Gm26659 | 2.729 | 2.32897 |
| Itfg1 | 2.72013 | 3.71283 |
| H2-Ea-ps | 2.71277 | 1.95307 |
| Rpl18 | 2.70007 | 1.13272 |
| Tspo | 2.67385 | 0.904701 |
| Gapdh | 2.67103 | 0.692244 |
| Tpt1-ps4 | 2.67043 | 4.70627 |
| Gm37945 | 2.66347 | 2.48272 |
| Gm17275 | 2.65444 | 1.84493 |
| Gnaq | 2.6535 | 2.25354 |
| Tcp1 | 2.65159 | 2.23636 |
| Rpl13 | 2.64703 | 0.752309 |
| Gpx4 | 2.64421 | 0.876561 |
| Sod2 | 2.64357 | 0.314076 |
| Emp3 | 2.64141 | 0.861668 |
| Gm43972 | 2.639 | 4.53148 |
| Slc28a1 | 2.63233 | 3.341 |
| Rps10 | 2.62613 | 1.46986 |
| Ifi204 | 2.62555 | 0.431153 |
| Gdi2 | 2.61952 | 0.544517 |
| Cfp | 2.6148 | 0.715343 |
| Rps3 | 2.61098 | 1.26913 |
| Gm44850 | 2.61042 | 0.701345 |
| Rap1b | 2.60522 | 0.918325 |
| Cox4i1 | 2.60522 | 1.42919 |
| Bst2 | 2.60205 | 0.515908 |
| Gm16755 | 2.59684 | 2.48249 |
| Cst3 | 2.59549 | 2.7613 |
| Gm42522 | 2.59113 | 1.23485 |
| Irf8 | 2.58919 | 0.549127 |
| Gbp5 | 2.58656 | 0.141689 |
| Sh3bgrl3 | 2.58642 | 1.09609 |
| Senp7 | 2.58351 | 1.52057 |
| Fau | 2.58335 | 1.39592 |
| Hs6st3 | 2.58196 | 4.79081 |
| Ifit1 | 2.5734 | 0.375328 |
| Gm38005 | 2.57266 | 1.66458 |
| B2m | 2.56687 | 1.79722 |
| Fpr2 | 2.56224 | 0.840314 |
| Trim30e-ps1 | 2.56058 | 0.817301 |
| Ccnd2 | 2.56034 | 0.136942 |
| Clec7a | 2.56033 | 1.64592 |
| Gm16505 | 2.55775 | 1.68433 |
| Rpsa | 2.54608 | 0.570002 |
| Grn | 2.54448 | 1.62052 |
| Samhd1 | 2.54305 | 0.199419 |
| Gm12352 | 2.54184 | 1.85558 |
| Aida | 2.53679 | 1.05258 |
| Gm13132 | 2.53551 | 1.5354 |
| Ldha | 2.53008 | 1.55545 |
| Rpl4 | 2.52956 | 0.350129 |
| Gpi1 | 2.515 | 0.197864 |
| RP24-83E15.3 | 2.50301 | 0.749867 |
| Ifi30 | 2.48837 | 0.21331 |
| EU599041 | 2.48169 | 2.3413 |
| Gm42641,Trex1 | 2.47941 | 0.340109 |
| Gm6421 | 2.47571 | 2.20461 |
| Phf11b | 2.47325 | 0.608203 |
| Hexa | 2.47121 | 0.839812 |
| Angel1 | 2.4624 | 5.2115 |
| Gm26809 | 2.46191 | 0.15887 |
| Adam8 | 2.4607 | 0.607344 |
| Alox5ap | 2.46007 | 0.168594 |
| Ccl17 | 2.45546 | 0.688799 |
| S100a11 | 2.45276 | 0.861777 |
| Tmsb4x | 2.45021 | 1.97058 |
| Gm38283 | 2.44685 | 1.28839 |
| Ucp2 | 2.44673 | 0.443519 |
| Myo9a | 2.43789 | 4.98087 |
| Gm2199 | 2.43525 | 1.80538 |
| Ctsa | 2.43354 | 1.38124 |
| Mx1 | 2.42968 | 0.112757 |
| Ass1 | 2.42666 | 0.646067 |
| Kctd12 | 2.42582 | 0.301817 |
| Ccr7 | 2.41951 | 2.2189 |
| Eno1 | 2.41747 | 0.726509 |
| Idh1 | 2.41565 | 0.245657 |
| Nfkb2 | 2.41393 | 0.18259 |
| RP24-229E19.1 | 2.4131 | 0.609477 |
| Gm16069 | 2.40894 | 0.446364 |
| Gm9575 | 2.40879 | 2.14355 |
| Psmb8 | 2.40849 | 1.12383 |
| Gm5809 | 2.40549 | 1.89847 |
| Gm44209 | 2.39827 | 1.96457 |
| Socs1 | 2.38805 | 0.193652 |
| Tpi1 | 2.38787 | 0.878939 |
| Rpl26 | 2.38429 | 1.0076 |
| Msn | 2.38396 | 0.341381 |
| Slc1a1 | 2.37644 | 3.23901 |
| Gm8545 | 2.37615 | 5.30664 |
| Garnl3 | 2.37528 | 1.31759 |
| Acyp2 | 2.3737 | 2.06903 |
| RP24-285L8.3 | 2.37015 | 1.3115 |
| Taldo1 | 2.35546 | 0.591207 |
| 1700021J08Rik | 2.35246 | 0.642729 |
| Chpt1 | 2.35053 | 1.99007 |
| Slc25a5-ps | 2.3409 | 3.45887 |
| Rpl34 | 2.33746 | 1.65785 |
| Gm4076 | 2.33421 | 1.69411 |
| Ch25h | 2.33063 | 0.245146 |
| Mmp9 | 2.32871 | 1.0011 |
| Chchd7 | 2.32804 | 2.08771 |
| Rnf19b | 2.3277 | 0.206804 |
| Fgl2 | 2.32547 | 0.531586 |
| Zfp36 | 2.32444 | 0.51626 |
| Gm42413 | 2.32206 | 0.66737 |
| H2-D1 | 2.31938 | 0.768421 |
| Gm2824 | 2.31127 | 2.69659 |
| Gm10698 | 2.30509 | 3.51958 |
| Nme1 | 2.29865 | 0.849249 |
| Rab7 | 2.29324 | 1.48912 |
| Csrp1 | 2.29303 | 0.568482 |
| Psma6 | 2.2843 | 0.927582 |
| Six2 | 2.26833 | 1.0947 |
| Ifitm3 | 2.26648 | 1.89849 |
| Gpr84 | 2.26395 | 0.236583 |
| Txnrd1 | 2.26186 | 1.39451 |
| Rpl7 | 2.25899 | 0.761295 |
| Arg1 | 2.25499 | 0.249424 |
| Ryr3 | 2.25486 | 2.63722 |
| Cyba | 2.25285 | 1.62561 |
| Ighv1-64 | 2.24522 | 1.45937 |
| H2-Q2 | 2.24498 | 0.834971 |
| Sepw1 | 2.24399 | 1.3067 |
| Gm17711 | 2.24238 | 1.58204 |
| Gm42959 | 2.23968 | 0.508458 |
| Cxcl14 | 2.2391 | 1.42987 |
| Mtpn | 2.23851 | 0.735174 |
| RP24-560O24.2 | 2.23637 | 0.325582 |
| Rpl3 | 2.22722 | 0.459069 |
| Rpl14 | 2.22271 | 0.529895 |
| Hspe1-ps6 | 2.22145 | 1.39595 |
| Inhba | 2.2199 | 0.214875 |
| Rnh1 | 2.21841 | 1.09841 |
| Gm15482 | 2.20991 | 1.75792 |
| Capg | 2.19887 | 2.22132 |
| Psap | 2.19847 | 2.76726 |
| Grid1 | 2.1969 | 1.24882 |
| Gm8801 | 2.18709 | 2.36182 |
| Irgm1 | 2.18336 | 0.457369 |
| Gm11867 | 2.17643 | 2.18132 |
| Gm20412 | 2.17279 | 0.188788 |
| Nt5c3 | 2.17174 | 0.58084 |
| Pgd | 2.16202 | 0.776179 |
| Gbx1 | 2.1533 | 2.23084 |
| Ybx1 | 2.1532 | 1.03657 |
| Sqstm1 | 2.15198 | 0.418388 |
| Wfdc17 | 2.15123 | 3.25141 |
| Gm21961 | 2.15052 | 2.6749 |
| Fam26f | 2.14915 | 0.547939 |
| Psmb10 | 2.14777 | 0.632228 |
| Prex2 | 2.13984 | 1.93291 |
| Rpl23a | 2.13221 | 0.88459 |
| Snx22 | 2.1305 | 1.10669 |
| Gm8221 | 2.1299 | 0.226555 |
| Tnfaip3 | 2.12924 | 0.1418 |
| Dusp1 | 2.12651 | 0.198317 |
| Gm30292 | 2.12619 | 1.59556 |
| Gm15932 | 2.12601 | 0.628304 |
| Zfp280c | 2.12208 | 4.36897 |
| Calr | 2.12167 | 2.01946 |
| Gm16407 | 2.11595 | 2.93881 |
| Gm43842 | 2.11536 | 0.20969 |
| Gm13453 | 2.11058 | 3.82685 |
| Cd69 | 2.11003 | 0.365441 |
| Gm29585 | 2.10914 | 0.951327 |
| Gm16234 | 2.10219 | 1.01388 |
| Slc25a5 | 2.10136 | 1.01487 |
| Gm44364 | 2.09728 | 1.26053 |
| Sh3bgrl | 2.09397 | 0.391375 |
| Cntnap2 | 2.08982 | 1.25241 |
| Calm2 | 2.08863 | 0.48895 |
| Rpl36a-ps1 | 2.08493 | 4.00681 |
| Calm1 | 2.0788 | 1.33098 |
| Cd63 | 2.07776 | 1.66504 |
| Itgb2 | 2.06671 | 1.54708 |
| Cfhr1 | 2.06652 | 0.910512 |
| Ctsh | 2.06629 | 0.657358 |
| Tgfbi | 2.06593 | 0.603445 |
| Gm6265 | 2.06386 | 2.85354 |
| Pcbp2 | 2.05896 | 0.533139 |
| Sepn1 | 2.05407 | 0.504427 |
| Gm26762 | 2.05016 | 0.543848 |
| Rpl17 | 2.04702 | 0.91954 |
| Gm8410 | 2.04313 | 1.50342 |
| Gm6788 | 2.03824 | 1.09526 |
| Gm43963 | 2.03564 | 0.337152 |
| Rpl7a | 2.03382 | 0.602058 |
| RP23-96L20.1 | 2.03109 | 3.38757 |
| Rps7 | 2.03052 | 0.852045 |
| RP23-457I3.2 | 2.03002 | 1.91737 |
| Pou3f4 | 2.02927 | 2.49551 |
| Ifitm1 | 2.02917 | 1.73622 |
| Ms4a4c | 2.02748 | 0.550882 |
| Rpl31 | 2.02461 | 0.896818 |
| Gadd45b | 2.02412 | 0.344203 |
| Gm18733 | 2.02229 | 0.338088 |
| Kxd1 | 2.02076 | 3.11027 |
| Tap1 | 2.01722 | 0.260297 |
| Rps29 | 2.01712 | 3.06822 |
| Eif5a | 2.01627 | 1.76561 |
| 5830462I19Rik | 2.01327 | 0.326901 |
| Snx5 | 2.00808 | 0.24989 |
| Chmp4b | 2.00709 | 0.348776 |
| Gm18367 | 2.00613 | 2.78277 |
| Rps13 | 2.00467 | 1.01123 |
| Gm26561 | 2.00016 **^4^** | 1.52978 |
|  |  |  |

**^1^** The value represents the difference between the distinct groups

**^2^** The value highlights variety within each sample group

**^3^** mRNA transcripts indicating immune function are shaded in blue, metabolism shaded in green and regulation shaded in orange

**^4^**The VIP list was limited to a VIP score ≥ 2.00

## Table 3. Pairwise analysis of metabolites between *T. gondii* infected and naïve BMDC cultures.

| Metabolite | M2.VIP[1+3+0]**^1^** | 2.57059 * M2.VIP[1]cvSE**^2^** |
| --- | --- | --- |
| Choline phosphate | 6.76561 | 1.69423 |
| Creatine | 5.8087 | 1.69916 **^3^** |
| L-Proline | 5.01163 | 2.40337 |
| sn-glycero-3-Phosphocholine | 4.73618 | 1.5894 |
| L-Glutamine | 3.50525 | 0.844527 |
| (R)-Lactate | 2.50828 | 0.988988 |
| 2-C-Methyl-D-erythritol 4-phosphate | 2.38283 | 1.99676 |
| L-Leucine | 2.33065 | 0.923908 |
| Creatinine | 2.12574 | 0.883704 |
| 5-Aminopentanoate | 2.02457 | 1.52706 |
| L-Glutamate 5-semialdehyde | 1.93553 | 0.31568 |
| L-1-Pyrroline-3-hydroxy-5-carboxylate | 1.64142 | 0.764856 |
| Hexadecanoic acid | 1.52687 | 1.25065 |
| Orthophosphate | 1.37493 | 0.806918 |
| 4-Aminobutanoate | 1.3434 | 0.127449 |
| L-Methionine | 1.32596 | 0.60832 |
| O-Acetylcarnitine | 1.27266 | 0.436977 |
| L-Phenylalanine | 1.24928 | 0.588447 |
| L-Ornithine | 1.19816 | 1.1703 |
| L-Arginine | 1.00167 | 0.670487 |
| (S)-Malate | 0.967498 | 0.13752 |
| L-Glutamate | 0.939875 | 0.122805 |
| Taurine | 0.935706 | 0.535007 |
| 4-Trimethylammoniobutanoate | 0.933063 | 0.509923 |
| Pyridoxine | 0.906283 | 0.421611 |
| Citrate | 0.849383 | 0.472178 |
| L-Carnitine | 0.829259 | 0.613708 |
| N6_N6_N6-Trimethyl-L-lysine | 0.812306 | 0.241484 |
| N-Acetyl-L-histidine | 0.809274 | 0.255653 |
| Glutathione | 0.808115 | 0.223587 |
| L-Threonine | 0.757361 | 0.104131 |
| Choline | 0.729203 | 0.196104 |
| L-Tyrosine | 0.698689 | 0.268397 |
| Itaconate | 0.69624 | 0.626784 |
| LL-2_6-Diaminoheptanedioate | 0.638327 | 0.263085 |
| L-Alanine | 0.635326 | 0.343015 |
| Guanidinoacetate | 0.568336 | 0.850776 |
| N(pi)-Methyl-L-histidine | 0.527641 | 0.285239 |
| Dimethisterone | 0.498988 | 0.515432 |
| L-Aspartate | 0.461348 | 0.0754728 |
| 4-Guanidinobutanoate | 0.406417 | 0.265405 |
| Imidazole-4-acetate | 0.337415 | 0.1142 |
| 5-Guanidino-2-oxopentanoate | 0.317114 | 0.0671731 |
| sn-glycero-3-Phosphoethanolamine | 0.294295 | 0.301633 |
| L-4-Hydroxyglutamate semialdehyde | 0.293947 | 0.162372 |
| Triethanolamine | 0.252115 | 0.704541 |
| L-Citrulline | 0.226159 | 0.0918497 |
| N2-(D-1-Carboxyethyl)-L-arginine | 0.114314 **^4^** | 0.0739838 |

## Table 4. Pairwise analysis of metabolites between LPS stimulated and naïve BMDC cultures.

| Metabolite | M1.VIP[1+2+0]**^1^** | 2.57059 * M1.VIP[1]cvSE**^2^** |
| --- | --- | --- |
| Creatine | 6.3808 | 3.18602 |
| sn-glycero-3-Phosphocholine | 5.79294 | 2.09949 |
| L-Proline | 4.78237 | 1.32636 **^3^** |
| L-Glutamine | 3.6562 | 1.21007 |
| 5-Aminopentanoate | 3.62745 | 4.36732 |
| (R)-Lactate | 3.07121 | 0.552185 |
| Choline phosphate | 2.32226 | 3.92437 |
| Hexadecanoic acid | 2.14372 | 1.9965 |
| L-Arginine | 2.04938 | 1.4134 |
| L-Leucine | 2.02796 | 0.759566 |
| L-Citrulline | 1.8282 | 0.268927 |
| (S)-Malate | 1.81556 | 0.70968 |
| L-1-Pyrroline-3-hydroxy-5-carboxylate | 1.79401 | 1.14217 |
| Citrate | 1.70937 | 0.432366 |
| O-Acetylcarnitine | 1.69219 | 1.17336 |
| L-Ornithine | 1.68615 | 0.649887 |
| 4-Trimethylammoniobutanoate | 1.65289 | 1.06466 |
| L-Carnitine | 1.63514 | 2.54921 |
| L-Glutamate | 1.32708 | 1.55263 |
| L-Glutamate 5-semialdehyde | 1.32572 | 0.474043 |
| Creatinine | 1.25666 | 1.07967 |
| Dimethisterone | 1.1949 | 0.926273 |
| L-Methionine | 1.14539 | 0.456413 |
| Itaconate | 1.13803 | 1.32046 |
| Guanidinoacetate | 1.09384 | 1.71369 |
| L-Phenylalanine | 1.07525 | 0.245463 |
| Taurine | 1.04082 | 1.57372 |
| 2-C-Methyl-D-erythritol 4-phosphate | 1.02918 | 1.79602 |
| L-Aspartate | 1.00358 | 0.30503 |
| Choline | 0.95457 | 1.16894 |
| Orthophosphate | 0.947722 | 1.40492 |
| N2-(D-1-Carboxyethyl)-L-arginine | 0.809032 | 0.0792187 |
| N6_N6_N6-Trimethyl-L-lysine | 0.80748 | 0.402302 |
| LL-2_6-Diaminoheptanedioate | 0.780014 | 0.261569 |
| Triethanolamine | 0.76807 | 1.95475 |
| L-Alanine | 0.763904 | 0.815921 |
| Imidazole-4-acetate | 0.731588 | 0.120605 |
| N-(omega)-Hydroxyarginine | 0.710251 | 0.154233 |
| L-Threonine | 0.708113 | 0.116189 |
| 4-Guanidinobutanoate | 0.698755 | 0.427768 |
| Glutathione | 0.688964 | 1.13186 |
| 4-Aminobutanoate | 0.663716 | 0.629633 |
| N-Acetyl-L-histidine | 0.613333 | 0.30308 |
| Pyridoxine | 0.596566 | 0.546327 |
| N(pi)-Methyl-L-histidine | 0.588798 | 0.242497 |
| sn-glycero-3-Phosphoethanolamine | 0.578397 | 0.266971 |
| L-Tyrosine | 0.554168 | 0.148588 |
| 5-Guanidino-2-oxopentanoate | 0.528328 | 0.0875291 |
| L-4-Hydroxyglutamate semialdehyde | 0.509195 **^4^** | 0.280166 |

**^1^** The value represents the difference between the distinct groups

**^2^** The value highlights variety within each sample group

**^3^** Metabolites from each pathway are highlighted in the VIP table where glycolysis is coloured green, TCA cycle; blue, OXPHOS; purple, arginine metabolism; orange and parasite specific metabolites; yellow.

**^4^** VIP score list (generated from OPLS-DA plot) was limited to 50 metabolites for the metabolomics studies.

**Table 5. High quality transcriptomic data was obtained for BMDC activated with LPS or co-cultured with *T. gondii* ^1^**

| **Sample** | **Total Reads** | **Discarded Reads** | **Clean Reads** | **Mapped Reads^2^** | **% Mapped** |
| --- | --- | --- | --- | --- | --- |
| UN1 | 46, 050, 349 | 0.4% | 45, 874, 907 | 44, 913, 488 | 97.9 |
| UN2 | 42, 960, 078 | 0.4% | 42, 770, 393 | 41, 849, 911 | 97.8 |
| UN3 | 51, 319, 651 | 0.4% | 51, 319, 651 | 50, 388, 676 | 98.2 |
| LPS1 | 40, 239, 286 | 0.4% | 40, 072, 860 | 39,229, 554 | 97.9 |
| LPS2 | 43, 550, 476 | 0.4% | 43, 388, 004 | 42, 273, 359 | 97.4 |
| LPS3 | 47, 700, 318 | 0.4% | 47, 517, 704 | 46, 701, 407 | 98.3 |
| Tg1 | 44, 610, 461 | 0.5% | 44, 401, 746 | 40, 497, 402 | 91.2 |
| Tg2 | 36, 114, 699 | 0.5% | 35, 937, 718 | 41, 849, 911 | 90.0 |
| Tg3 | 43, 868, 055 | 0.5% | 43, 660, 931 | 50, 388, 676 | 88.5 |

^1^Bone marrow derived dendritic cells were stimulated with LPS or infected with *L. mexicana* or *T. gondii,* 6 hours before mRNA was extracted.

^2^Clean reads were mapped to the *Mus musculus* reference genome.

**Amino acid metabolism**

**Figure 1. Global metabolic activity of BMDCs co-cultured with *T. gondii*or stimulated with LPS.**Bone marrow derived DCs were co-cultured with *T. gondii* or stimulated with LPS as indicated for 24 hours. After this time, the metabolites of the BMDCs were extracted and measured via Liquid chromatography mass spectroscopy (LCMS).  A heat map was constructed from the analysed data on IDEOM and Prism7 and shows the fold change increase (Red) or the decrease (Green) of each metabolite compared to its representative unstimulated control (Grey). Standards are highlighted in bold. N = 3.

**Carbohydrate Metabolism**

**Nucleotide Metabolism**

**Amino Acid Metabolism**

**Figure 2. Global metabolic activity of BMDCs co-cultured with PFA fixed *T. gondii*or treated with TLA.**Bone marrow derived DCs were co-cultured with *T. gondii* or treated with TLA as indicated for 24 hours. After this time, the metabolites of the BMDCs were extracted and measured via Liquid chromatography mass spectroscopy (LCMS).  A heat map was constructed from the analysed data on IDEOM and Prism7 and shows the fold change increase (Red) or the decrease (Green) of each metabolite compared to its representative unstimulated control (Grey). Standards are highlighted in bold. N = 1.

**
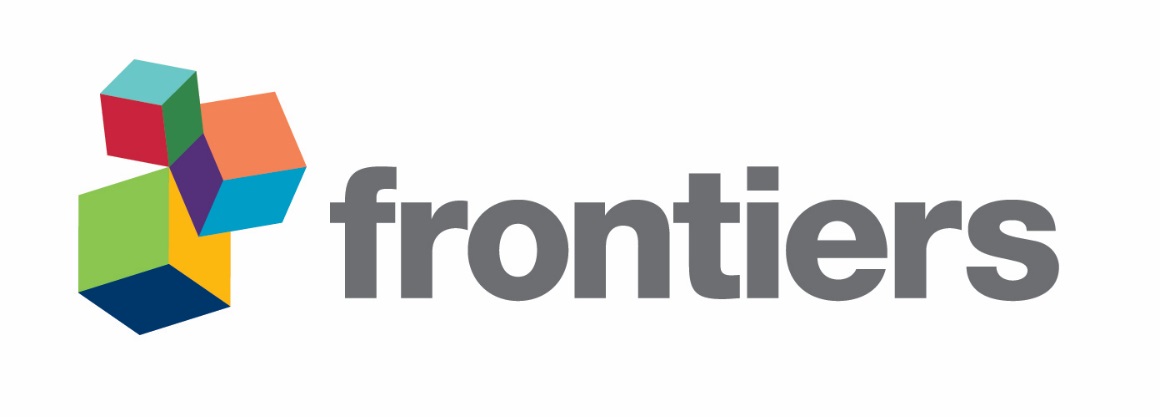
**
